# Transcriptome and Lipidomic Analysis Suggests Lipid Metabolism Reprogramming and Upregulating *SPHK1* Promotes Stemness in Pancreatic Ductal Adenocarcinoma Stem-like Cells

**DOI:** 10.3390/metabo13111132

**Published:** 2023-11-04

**Authors:** Jinzhi Xu, Lina Zhou, Xiaojing Du, Zhuoran Qi, Sinuo Chen, Jian Zhang, Xin Cao, Jinglin Xia

**Affiliations:** 1 National Medical Center and National Clinical Research Center for Interventional Medicine, Liver Cancer Institute, Zhongshan Hospital, Fudan University, 180 Fenglin Road, Shanghai 200032, China; 2 CAS Key Laboratory of Separation Science for Analytical Chemistry, Dalian Institute of Chemical Physics, Chinese Academy of Sciences, Dalian 116023, China; 3 Endoscopy Center, Shanghai East Hospital, Tongji University School of Medicine, Shanghai 200120, China; 4 Institute of Clinical Science, Zhongshan Hospital, Fudan University, Shanghai 200032, China; 5 Key Laboratory of Diagnosis and Treatment of Severe Hepato-Pancreatic Diseases of Zhejiang Province, The First Affiliated Hospital of Wenzhou Medical University, Wenzhou 325000, China

**Keywords:** pancreatic ductal adenocarcinoma, cancer stem-like cells, lipid metabolism reprogramming, sphingolipid metabolism, *SPHK1*

## Abstract

Cancer stem cells (CSCs) are considered to play a key role in the development and progression of pancreatic ductal adenocarcinoma (PDAC). However, little is known about lipid metabolism reprogramming in PDAC CSCs. Here, we assigned stemness indices, which were used to describe and quantify CSCs, to every patient from the Cancer Genome Atlas (TCGA-PAAD) database and observed differences in lipid metabolism between patients with high and low stemness indices. Then, tumor-repopulating cells (TRCs) cultured in soft 3D (three-dimensional) fibrin gels were demonstrated to be an available PDAC cancer stem-like cell (CSLCs) model. Comprehensive transcriptome and lipidomic analysis results suggested that fatty acid metabolism, glycerophospholipid metabolism, and, especially, the sphingolipid metabolism pathway were mostly associated with CSLCs properties. *SPHK1* (sphingosine kinases 1), one of the genes involved in sphingolipid metabolism and encoding the key enzyme to catalyze sphingosine to generate S1P (sphingosine-1-phosphate), was identified to be the key gene in promoting the stemness of PDAC. In summary, we explored the characteristics of lipid metabolism both in patients with high stemness indices and in novel CSLCs models, and unraveled a molecular mechanism via which sphingolipid metabolism maintained tumor stemness. These findings may contribute to the development of a strategy for targeting lipid metabolism to inhibit CSCs in PDAC treatment.

## 1. Introduction

Pancreatic cancer is one of the most fatal cancers, ranking the seventh leading cause of cancer-related deaths worldwide [1], and has been predicted to become the second most common cause of cancer-related deaths by 2030 [2]. As the most common histological type, pancreatic ductal adenocarcinoma (PDAC) accounts for the majority of the incidence and mortality of pancreatic cancer cases [3]. Its strong heterogeneity endows PDAC with the feature of high lethality, which is thought to be closely related to a small group of cells that are characterized by self-renewal, unique plasticity and metabolism, and high proliferative capacity [4,5], known as cancer stem cells (CSCs) [6,7]. Therapy resistance of PDAC CSCs [8] is also mainly responsible for the limited survival benefit of chemotherapeutic agents, targeted therapy and immunotherapy for PDAC patients [9,10]. Thus, an in-depth understanding of CSCs in PDAC is urgently needed and may provide a foundation to explore new therapeutic strategies for clinical practice.

Metabolic reprogramming is one of the major hallmarks of tumorigenesis [11]. PDAC cells rely on altered metabolism pathways, including enhanced aerobic glycolysis [12,13], deregulation of lipid metabolism [14,15], raised branched-chain amino acids and glutamine routes [16,17], and increased nucleotide metabolism [18,19], to support their unlimited proliferation and metastasis [20]. Recent studies suggest that due to the heterogeneity of tumors, unique metabolism characteristics play a distinctive role in maintaining the pluripotency and tumorigenic capacity in PDAC CSCs [21,22]. PDAC CSCs are supposed to facilitate the metabolic flip from glycolytic to oxidative [21,23]. In addition, glutamine dependence is not limited to PDAC cells, as CSCs also rely on glutamine metabolism to promote tumor growth [24,25]. It is well acknowledged that CSCs can reprogram their cellular metabolism to support their continuous proliferation and tumorigenesis [20,26,27], while the understanding of the lipid metabolism disorder in CSCs is limited and unilateral. Several lipid metabolites [28,29] or lipid-metabolism-related genes (LMRGs) [30,31,32] have separately been reported to play important roles in maintaining the stemness and enhancing tumor metastasis. It is worth noting that tumor metabolic remodeling is a dynamic process; thus, studying changes in metabolic pathways may be more appropriate than directly studying the role of specific metabolite differences. Observed changes due to stemness acquisition in CSCs often encompass large numbers of structurally related lipids, and recent developments in technologies, such as lipidomic and machine learning, enable researchers to explore the lipid metabolism pathways and more comprehensively underline altered lipid metabolism in tumorigenesis [33]. In an attempt to explain the lipid metabolism characteristics of PDAC CSCs, only one institution has carried out a proteomic analysis and subsequently a comprehensive proteomic and lipidomic report on pancreatic cancer stem-like cells (CSLCs). They have reported that fatty acid synthesis, especially biosynthesis of unsaturated FAs, and mevalonate pathways, with downregulation of LDHA (Lactate Dehydrogenase A) and upregulation of genes involved in FA elongation, are essential in PDAC CSLCs [34,35]. Despite multi-omics characterization of PDAC CSCs suggesting the importance of lipid metabolic alterations, explorations on further characterization are still deficient. On the one hand, studies of lipid metabolism in PDAC CSCs were performed only on one traditional CSCs model. On the other hand, it appears that CSCs are extremely reliant on the enzymes involved in the lipid metabolism, but there is currently no research on transcriptomics that combines the stemness phenotypes of the patient’s tumor and the CSCs model.

In this study, we explored the difference in the lipid metabolism in patients with high and low stemness indices using single sample gene set enrichment analysis (ssGSEA) algorithms based on the data from the Cancer Genome Atlas (TCGA). Then, to overcome the limitation of the traditional CSCs model by sorting CSC of PDAC based on the identified “stem cell surface markers”, we used 3D soft fibrin-gel as the culture medium to select malignant tumor cells with high tumorigenicity in PDAC by adjusting the mechanical stress, defined as tumor-repopulating cells (TRCs), which has been successfully applied in many tumors, such as liver cancer, melanoma, lung adenocarcinoma, etc. [36,37]. Lipidomic combined with transcriptome analysis has been carried out and the results suggested that fatty acid metabolism, sphingolipid metabolism and glycerophospholipid metabolism alterations were mostly observed in PDAC TRCs. Further investigations revealed that *SPHK1*, encoding the key enzyme SPHK1 (sphingosine kinases 1) to catalyze sphingosine to generate S1P (sphingosine-1-phosphate) in sphingolipid metabolism, contributed to promote the stemness of PDAC, which may be a promising therapeutic target in PDAC.

## 2. Materials and Methods

### 2.1. Patients’ Data Collection and Analysis

The mRNA expression profiles and clinical features of PDAC patients were downloaded from the TCGA data portal (https://portal.gdc.cancer.gov/, accessed on 20 November 2022). The stemness indices were assigned to every PDAC patients from TCGA (tumor, *n* = 179), which were calculated by using ssGSEA algorithms [38] and one-class logistic regression machine learning (OCLR) algorithms [39]. The stemness gene set was obtained from Miranda’s studies and applied to the ssGSEA algorithm to calculate ssGSEA-based stemness indices (Table S1) [38]. The mean (the standard error of the mean (SEM)) of the stemness indices using the ssGSEA algorithm was 2.069 (0.011), and patients with stemness indices of less than 2.058 (mean-SEM) and over 2.080 (mean + SEM) were classified into the low stemness group and the high stemness group, respectively. PDAC is formally staged using a tumor node metastasis (TNM) system based on the eighth edition of the American Joint Committee on Cancer Staging Manual [40]. Student’s t-test was used to assess the relationship of clinical information and stemness indices and the results were plotted using the “ggplot2” (http://cran.r-project.org/package=ggplot2, accessed on 25 November 2022) package. The Kaplan–Meier (K–M) curve was plotted using the “survival” package (https://cran.r-project.org/package=survival, accessed on 25 November 2022) to achieve the survival analysis of patients with high or low stemness indices. The “DESeq2” R package was employed to identify the differential expressed genes (DEGs) between patients with high or low stemness indices. Enrichment analysis of the DEGs was conducted as follows.

### 2.2. Enrichment Analysis

Kyoto Encyclopedia of Genes and Genomes (KEGG) and Gene Ontology (GO) analyses were performed by using the “clusterProfiler” [41] package of R and visualized by applying the “ggplot2” package. Enrichment gene sets, including c2.cp.kegg.v7.4.symbols and h.all.v7.5.1.symbols, were obtained from the Molecular Signatures Database (MSigDB) [42]. Gene set variation analysis (GSVA) was utilized to calculate the enrichment score of these oncogenic signatures [43]. The correlation between SPHK1 and pathway scores was analyzed via Spearman correlation. The “pheatmap” R package was used for clustering heatmaps with standardization processing “scale = row”.

### 2.3. Cell Line and Cell Culture

PDAC cell lines (MiaPaCa-2, PANC−1) were obtained from the American Type Culture Collection (ATCC) and were preserved at the Liver Cancer Institute, Zhongshan Hospital, Fudan University (Shanghai, China). All cells passed conventional quality control tests, which was consistent with the findings reported by the ATCC. The culture conditions for the cell lines were complete medium, consisting of Dulbecco’s modified Eagle’s medium (DMEM; GNM12800-2, GENOM, Jiaxing, Zhejiang, China) supplemented with 10% fetal bovine serum (FBS; 10270-106, Gibco, Grand Island, NY, USA) and 1% penicillin-streptomycin (1719675, Gibco, Grand Island, NY, USA), in a humidified ThermoForma incubator (Thermo Fisher Scientific, Waltham, MA, USA) with 37 ℃ and 5% CO_2_, as described in a previous study [44].

### 2.4. Culture of PDAC TRCs

A previous study showed that TRCs cultured in the 3D fibrin gels represented an available CSLCs [37]. Thus, we cultured PDAC-TRCs as previously described [36]. Specifically, MiaPaCa-2 or PANC−1 cells were trypsinized and resuspended in complete medium, and then mixed with an equal volume 2 mg/mL salmon fibrinogen (SEA-133, Sea Run Holdings Inc., Freeport, ME, USA) diluted with T7 buffer (50 mM Tris-HCl, 150 mM NaCl, pH 7.4). Next, 100 U/mL thrombin (SEA-135, Sea Run Holdings Inc., Freeport, ME, USA) diluted with T7 buffer was added at a 1:50 ratio to the cell suspension to form cell mixture. The complete medium was added to cell plates after incubation for 30 min in a humidified ThermoForma incubator (Thermo Fisher Scientific, Waltham, MA, USA) with 37 °C and 5% CO_2_, and the cells were sequentially cultured for 72 h. The resulting PDAC-TRCs were used for subsequent experiments.

### 2.5. Quantitative Reverse Transcription Polymerase Chain Reaction (qRT-PCR)

The mRNA of 2D−cultured cells or PDAC TRCs was extracted by using an RNAeasy^TM^ kit (R0026; Beyotime Biotechnology, Shanghai, China) according to the manufacturer’s recommended procedure, and then reversely transcribed into cDNA by using Hifair^®^ V one-step RT-gDNA digestion SuperMix Kit (11141ES60, Yeasen, Shanghai, China) according to manufacturer’s instructions. Then, the conditions of qRT−PCR were set as follows: initial denaturation at 95 °C for 5 min; and 40 cycles of 95 °C for 10 s and 60 °C for 30 s, which was performed using SYBR Green kit (11202ES08) on QuantStudio5 fluorescence quantitative PCR system (Applied Biosystems, Foster City, CA, USA). The sequences of all primers were displayed in Table 1. The 2^ΔΔCT^ method was used to calculate the relative gene expression change with β-actin as the internal normalization. Each experiment was performed with three independent replicates, and the results were displayed as the mean ± SD.

### 2.6. RNA Interference

To silence the expression of *SPHK1*, the cells were transfected with siRNA by applying riboFECT^TM^ CP (C10511-05, RIBOBIO, Guangzhou, Guangdong, China). The targeted sequence of siSPHK1 was GAGGCUGAAAUCUCCUUCATT.

### 2.7. Western Blotting

Western blotting was performed as described in our previous study [45]. Rabbit monoclonal to SPHK1((ab302714)) was purchased from Abcam.

### 2.8. Transwell Assays

Transwell assays were used to assess the invasion and migration ability of PDAC TRCs. For migration assays, 10,000 cells were placed into the upper chamber with DMEM medium, while for invasion assays, 10,000 cells were plated into the upper chamber, which was precoated with Matrigel (356234, BD Biosciences, San Jose, CA, USA) diluted at 1:8 with DMEM medium. Then, 800 μL of DMEM medium containing 20% FBS was added to the lower chamber and the cells were cultured for 48 h. Then, the cells in the upper chamber were carefully removed. The cells passing through the membrane filter were stained with 0.1% crystal violet solution (V5265, Sigma, St. Louis, MO, USA) and recorded by using a microscope and counted using Image J software (National Institutes of Health, Bethesda, MD, USA). Each experiment was performed with three independent replicates, and the results were displayed as the mean ± SD.

### 2.9. Reagent and Intervention Process

S1P (HY-108496) was purchased from MCE. The preparation of stock solution and storage were conducted according to the manufacturer’s recommended procedure. When PDAC TRCs transfected with si-SPHK1 were cultured in 3D gel for 5 days, exogenous S1P (10 μM) was supplemented and the growth of TRCs was continuously observed. In the Transwell assays, 10 μM S1P was added to the lower chamber in the testing group.

### 2.10. Subcutaneous Tumors in Mice

Four-week-old male nude (*nu*/*nu*) mice were obtained from the Shanghai Institute of Material Medicine (Shanghai, China), Chinese Academy of Science. All mice were randomly allocated to 2D group or TRC group (*n* = 18 for each group). For subcutaneous tumors, single-cell suspensions of PANC−1 and PANC−1 TRCs were injected with gradient cell density (2 × 10^4^, 2 × 10^5^, 2 × 10^6^, *n* = 6 for every group) on the right side of the armpit of the nude mice. The animal study protocols were performed in accordance with the Guide for the Care and Use of Laboratory Animals stipulated by the National Academy of Sciences and the National Institutes of Health (NIH publication 86-23, revised 1985) and approved by the Animal Care and Use Committee of Zhongshan Hospital, Fudan University, Shanghai, China (Approval No. 2020-135 and date of approval 2 November 2020).

### 2.11. RNA-Seq

The total RNA of 2D−cultured cells or PDAC TRCs (three replicates for each cell type) was extracted by using TRIzol reagent (Invitrogen, Carlsbad, CA, USA) and the quantity and purity were monitored using NanoDrop ND-1000 (NanoDrop, Wilmington, DE, USA) as well as Bioanalyzer 2100 (Agilent, Santa Clara, CA, USA). OligodT-magnetic-beads (25-61005, Thermo Fisher, Waltham, CA, USA)-enriched mRNAs were fragmented. cDNAs were synthesized from the fragmented RNA using a Reverse Transcriptase (Invitrogen SuperScript™ II Reverse Transcriptase, Carlsbad, CA, USA), and then sequenced using Illumina Novaseq™ 6000 (LC Bio Technology Co., Ltd., Hangzhou, Zhejiang, China). The obtained RNA-Seq raw data were uploaded to the Sequence Read Archive (SRA) database of the National Center for Biotechnology Information (NCBI) (https://www.ncbi.nlm.nih.gov/, accessed on 20 October 2023) with the accession number PRJNA-1020096.

### 2.12. The Procedure for LC-MS-Based Lipidomic Analysis

The samples of 2D−cultured PANC−1 cells or PANC−1 TRCs (four biological replicates for each cell type) were collected and were added into 1 mL of pre-cooled methanol with an internal standard (1 μg/mL of tridecanoic acid and n-valine). After vortexing for 1 min, the mixtures were stored at −80 °C ThermoForma incubator (Thermo Fisher Scientific, Waltham, MA, USA). Then, the sample preparation, lipidomic data acquisition, data preprocessing, and peak annotation were performed as described in our previous study [46].

### 2.13. Bioinformatics Analysis of Lipidomic Data

The lipid profile levels obtained above were loaded into an open access tool BioPAN, on LIPID MAPS Lipidomic Gateway (https://lipidmaps.org/biopan/, accessed on 25 July 2022) [47]. BioPAN calculates statistical scores for all possible lipid pathways to predict which are active or suppressed in PANC−1-TRCs samples compared to the PANC−1 cells samples. In brief, BioPAN workflow utilizes Z-score, which takes into account both the mean and the standard deviation to assume normally distributed data of lipid subclasses and determines a reaction or pathway to be significantly modified at a *p*-value  <  0.05 (equivalent to Z-score > 1.645). The calculation of the Z-score was detailed by Gaud et al. [48].

### 2.14. Statistical Analysis

All plots and statistical analyses were conducted using R 4.3.1 and GraphPad Prism 9.5.0. Student’s *t*-tests (two-tailed) and one-way analysis of variance (ANOVA) were used to compare the means of two or more samples. The predictable value of SPHK1 expression was assessed using univariate and multivariate Cox analysis. As for the cellular experiments, each experiment was performed with at least three independent replicates, and the results are displayed as the mean ± SD. A *p*-value of less than 0.05 was considered statistically significant, unless otherwise indicated. *, *p* < 0.05; **, *p* < 0.01; ***, *p* < 0.001; ****, *p* < 0.0001; ns, not significant.

## 3. Results

### 3.1. Correlation between Stemness Indices via ssGSEA Algorithms and Clinicopathological Characteristics of PDAC Patients

**Figure 1:**
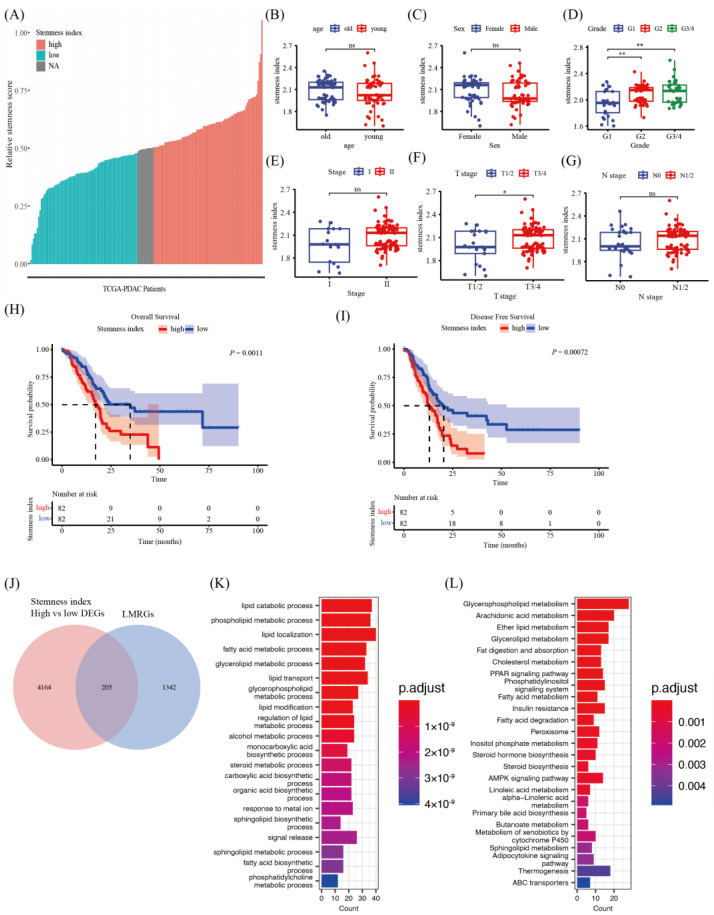
Correlation between stemness indices using ssGSEA and clinical features in PDAC patients. (**A**) An overview of the distribution of relative stemness indices in PDAC patients (*n* = 179) and the classification of stemness groups (high stemness > mean + SEM, *n* = 82; low stemness < mean—SEM, *n* = 82; NA~ mean ± SEM, *n* = 15). (**B**–**G**) Boxplots of stemness indices for PDAC patients stratified by clinical features including age, sex, pathological grade of tumor (2 Gx removed), N stage (5 Nx removed), T stage (2 Tx removed) and TNM stage (8 NA removed). OS K-M curve (**H**) and DFS K-M curve (**I**) showed the outcomes of PDAC patients in the high stemness group and low stemness group. (**J**) Venn diagram shows the overlapped genes between LMRGs and DEGs of the two stemness groups. (**K**) GO and (**L**) KEGG enrichment analysis of the overlapped genes. *, *p* < 0.05; and **, *p* < 0.01; Student’s *t*-test. ssGSEA, single sample gene set enrichment analysis; PDAC, pancreatic ductal adenocarcinoma; DFS, disease-free survival; K-M curve, Kaplan–Meier curve; OS, overall survival; LMRGs, lipid-metabolism-related genes; DEGs, differential expressed genes; GO, gene ontology; KEGG, Kyoto Encyclopedia of Genes and Genomes.

At first, we calculated the stemness indices using ssGSEA algorithms for each patient in TCGA-PDAC patients (*n* = 179) using RNA-seq data (Table S1). Then, according to the stemness indices, we ranked the patients from low to high (Figure 1A) and investigated the relationship between the indices and clinicopathological features including age, sex, pathological grade of tumor, N stage, T stage and TNM stage (Figure 1B–G). The results showed that patients with a higher pathological grade (Figure 1D, G2 vs. G1, *p* = 0.0018, and G3 vs. G1, *p* = 0.0016) or patients diagnosed with a higher T stage (Figure 1F, T3/4 vs. T1/2, *p* = 0.039) had significantly higher stemness indices. Moreover, the patients were divided into high (*n* = 82) and low stemness groups (*n* = 82) according to the aforementioned method, and survival analysis was conducted to compare these two groups. The K-M curve results showed that patients in the high stemness group suffered shorter median OS (high stemness group vs. low stemness group, 17.0 vs. 34.8 months, *p* = 0.0011, Figure 1H) and median DFS (high stemness group vs. low stemness group, 13.1 vs. 20.4 months, *p* = 0.0007, Figure 1I). Cox multivariate analysis with significant factors obtained from the univariate analysis (*p* < 0.05) was carried out to further assess the relationship between tumor stemness and patients’ OS (Table 2) and it was found that patients belonging to the low stemness group was an independent favorable prognosis factor for PDAC (HR = 0.594, 95% CI, 0.379–0.932, *p* = 0.023). However, the stemness indices using OCLR algorithms (Table S2) were not associated with patients’ OS (*p* = 0.15) and tumor dedifferentiation, as reflected in the histopathological grade (Figure S1). Taken together, these data suggested that the stemness indices using the ssGSEA algorithms could effectively distinguish PDAC patients and were consistent with the degree of tumor dedifferentiation and prognosis. Thus, we assumed that these stemness indices could be used to better describe and quantify CSCs in patients’ tumors.

### 3.2. Difference in Lipid Metabolism in Patients with High and Low Stemness Indices

More and more studies have shown that the dysregulation of lipid metabolism may be one of the most unique metabolic hallmarks of cancer, providing important targets for therapeutic interventions. To comprehensively elucidate the functional roles of deregulated lipid metabolic genes in PDAC patients, we selected 1543 LMRGs whose GO annotations included lipid-metabolism-related pathways. About 4.7% (205/4369) DEGs between tumors with high (*n* = 82) and low (*n* = 82) stemness indices are LMRGs (Figure 1J). To understand the characteristics of the lipid metabolism of PDAC, GO and KEGG enrichment analysis (Figure 1K,L and Tables S3 and S4) were performed and several specific genes and lipid metabolic pathways were identified, including fatty acid metabolism, glycerolipid metabolism, glycerophospholipid metabolism, and sphingolipid metabolism, etc. (Tables S3 and S4).

### 3.3. Characteristics of PDAC TRCs as an Available CSLCs Model

**Figure 2:**
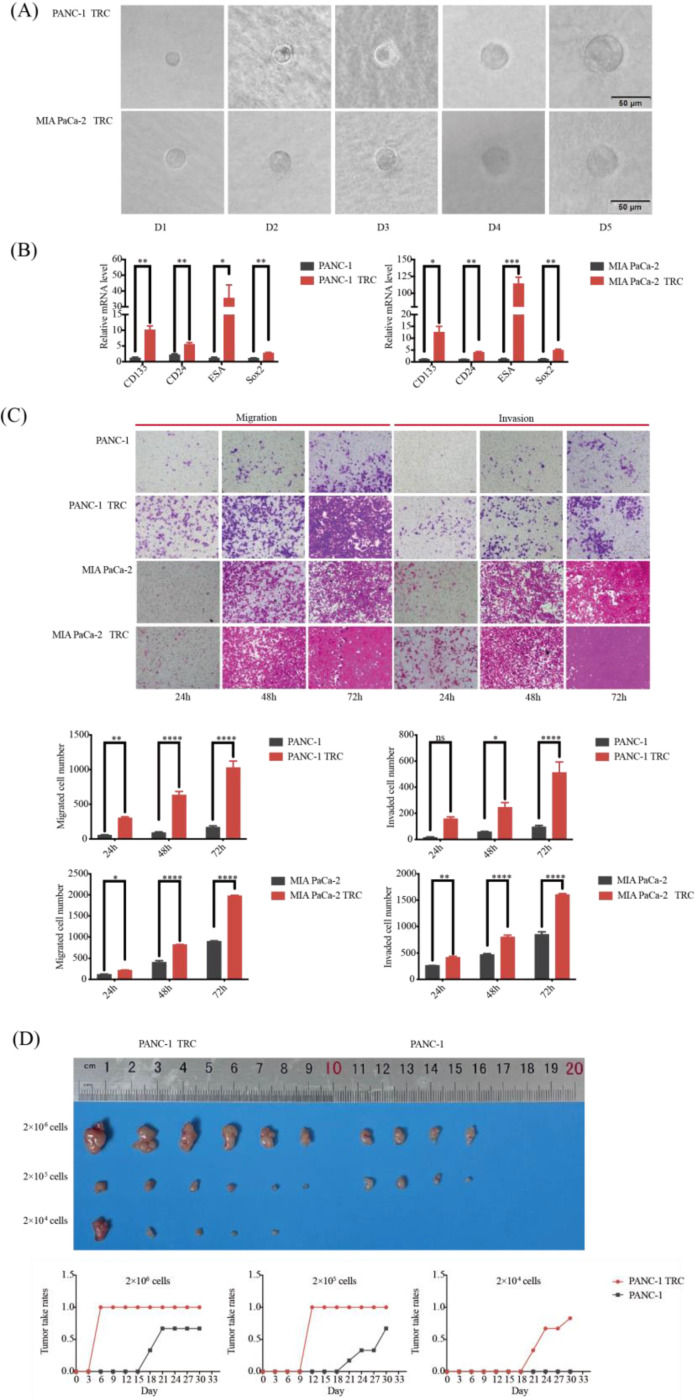
CLSCs’ characteristics of PDAC TRCs. (**A**) Morphology of PDAC TRCs growing in 3D soft gel fibers at days 1–5. (**B**) q-RT PCR showed expression of CSCs surface markers in PDAC TRCs and normal PDAC cells (mean ± SD, *n* = 3, *t*-test). (**C**) Transwell experiment showed migration and invasion abilities of PDAC TRCs and normal PDAC cells (mean ± SD, *n* = 3, *t*-test). (**D**) Morphology of subcutaneous tumors and tumorigenesis ability in nude mice of PDAC TRCs and normal PDAC cells (*n* = 6 for every gradient cell density in each cell type). *, *p* < 0.05; **, *p* < 0.01; ***, *p* < 0.001; and ****, *p* < 0.0001; ns, not significant. CLSCs, cancer stem-like cells; TRCs, tumor-repopulating cells; ESA, erythropoiesis-stimulating agent; Sox2, sex-determining region Y-box 2; q-RT PCR, quantitative reverse transcription polymerase chain reaction.

In order to better study the characteristics of the lipid metabolism of PDAC CSCs, we cultured human PDAC cell lines in 3D soft fiber gel to obtain PDAC TRCs, PANC−1 TRCs and MIA PaCa−2 TRCs, based on the method that our research team has previously confirmed to culture CLSCs in other tumor species [36,37]. PANC−1 TRCs and MIA PaCa−2 TRCs gradually formed clone spheres in 3D soft fiber gel, and the morphological changes from day 1 to day 5 are shown in Figure 2A. The qRT-PCR experiment results showed a significant increase in the expression of classic CSC surface markers CD133, CD24, ESA, and Sox2 in PANC−1 TRCs and MIA PaCa−2 TRCs compared to PANC−1 and MIA PaCa−2, respectively (Figure 2B). The transwell assays showed that PANC−1 TRCs and MIA PaCa−2 TRCs migrated to and invaded the lower chamber earlier than PANC−1 and MIA PaCa−2, and PANC−1 TRCs and MIA PaCa−2 TRCs exhibited more cell migration and invasion than their control groups within the same period of time (Figure 2C). These results proved that PANC−1 TRCs and MIA PaCa−2 TRCs captured a stronger tumorigenesis and metastasis ability than PANC−1 and MIA PaCa−2 in vitro.

In order to verify the malignant biology of TRCs in vivo, we constructed a subcutaneous tumor model in nude mice. PANC−1 cells and PANC−1 TRCs were inoculated with gradient cell density on the right side of the armpit near the back of the nude mice, and the tumorigenesis was observed daily. As shown in Table 3, the tumorigenesis rates of PANC−1 TRCs reached 83.3% at one month, while no tumor was observed in the 2 × 10^4^ PANC−1 group (Figure 2D and Figure S2A). Moreover, it was observed that the tumor formation time was earlier, and the tumor volume in the PANC−1 TRC group was larger after the same observation time (30 days) (Figure 2D and Figure S2B), further confirming the notable self-renewal and tumorigenic properties of PDAC TRCs.

### 3.4. Identification of Lipid Metabolism Pathways in PDAC TRCs via RNA-seq

**Figure 3:**
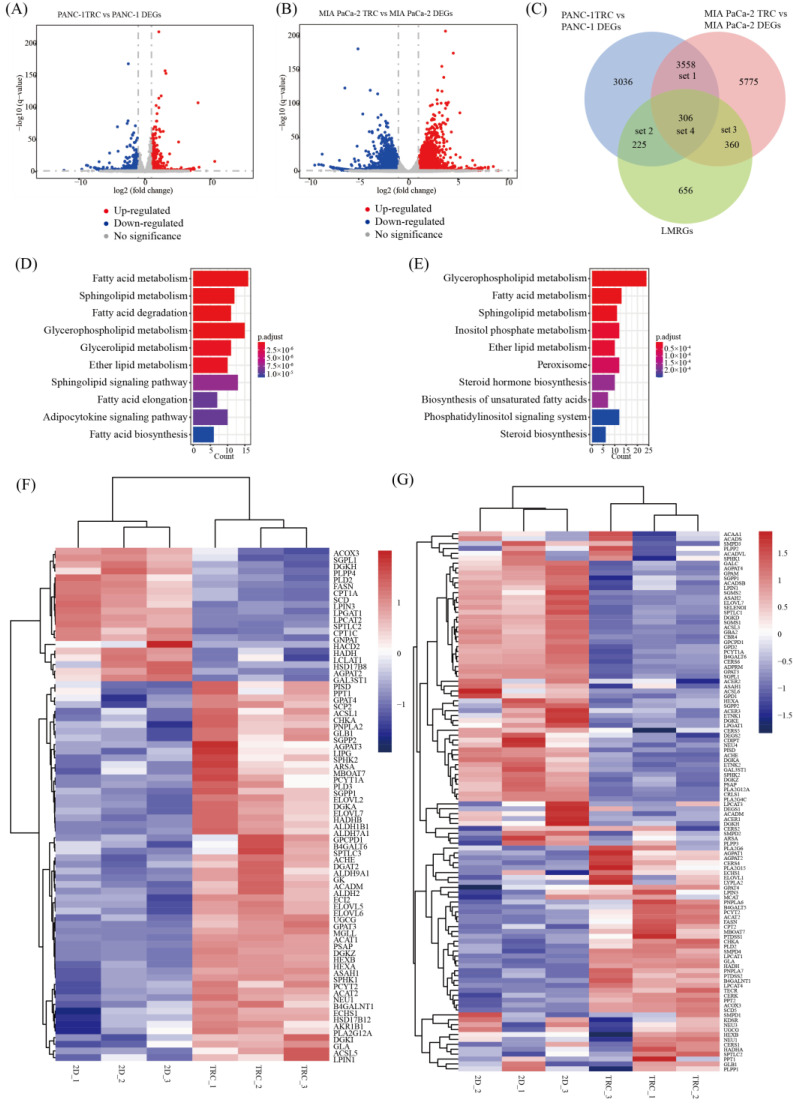
Identification of lipid metabolism reprogram pathways in PDAC TRCs via transcriptome analysis. (**A**) Volcano plots of DEGs of PANC−1 and PANC−1 TRCs (up-regulated genes in red and down-regulated in blue, *n* = 7125). (**B**) Volcano plots of DEGs of MIA PaCa−2 and MIA PaCa−2 TRCs (up-regulated genes in red and down-regulated in blue, *n* = 9999). (**C**) Venn diagram shows the overlapped genes between LMRGs and the DEGs of TRCs and the source normal 2D cells. Genes in set 2 (*n* = 531) were the LMRGs among DEGs of PANC−1 and PANC−1 TRCs and genes in set 3 (*n* = 666) were the LMRGs among DEGs of MIA PaCa−2 and MIA PaCa−2 TRCs. Genes in set 4 (*n* = 306) represented the overlapped genes between LMRGs set and the set 1 (common DEGs of PDAC TRCs and normal 2D−cultured cells, *n* = 3864) (**D**) Top 10 entries in KEGG enrichment pathway of genes in set2. (**E**) Top 10 entries in KEGG enrichment pathway of genes in set 3. (**F**) Heatmap of genes enriched in top 3 entries in KEGG enrichment pathway of genes in set 2 (relative high expression in red and relative low expression in blue). (**G**) Heatmap of genes enriched in top 3 entries in KEGG enrichment pathway of genes in set 3 (relative high expression in red and relative low expression in blue). PDAC, pancreatic ductal adenocarcinoma; TRCs, tumor-repopulating cells; DEGs, differential expressed genes; LMRGs, lipid-metabolism-related genes.

Since PDAC TRCs were proved to present CSCs features, PANC−1 TRCs and MIA PaCa−2 TRCs were used to explore the lipid metabolism characteristics of PDAC CSCs in gene expression level. About 7125 DEGs between PANC−1 and PANC−1 TRCs, as well as 9999 DEGs between MIA PaCa−2 and MIA PaCa−2 TRCs, were detected via RNA-seq (Figure 3A,B). As shown in Figure 3C, genes in set 2 (*n* = 531) were the LMRGs among DEGs of PANC−1 and PANC−1 TRCs, and genes in set 3 (*n* = 666) were the LMRGs among DEGs of MIA PaCa−2 and MIA PaCa−2 TRCs. Genes in set 4 (*n* = 306) represented the overlapped genes between LMRGs set and the set 1 (common DEGs of TRCs and normal 2D−cultured cells, *n* = 3864). The top three altered KEGG pathways of PANC−1 TRCs LMRGs (Figure 3D and Table S5) were fatty acid metabolism, sphingolipid metabolism and fatty acid degradation pathways, and the related LMRGs’ expression is shown in Figure 3F. When comparing MIA PaCa−2 TRCs with MIA PaCa−2, the top three altered KEGG pathways (Figure 3E and Table S6) were the glycerophospholipid metabolism, fatty acid metabolism and sphingolipid metabolism pathways, and the related LMRGs’ expression is shown in Figure 3G. It was obvious that fatty acid metabolism, and sphingolipid metabolism were commonly detected as the most altered pathways. Overall, the lipid metabolism pathways’ alteration in PDAC TRCs in different cell lines was similar, and the involved LMRGs may be different types of one genotype. In addition, the overlap of DEGs (set 4, *n* = 306, Figure 3C) with consistent trends in the two cell lines was analyzed for KEGG enrichment, and the results showed that glycerolipid metabolism, fat acid degradation, and sphingolipid metabolism pathways were the most significant changes in the lipid metabolism pathways (Figure S3 and Table S7). Despite numerous DEGs and lipid metabolic modifications in the common consistent trends set or between individual cell lines, each of our analysis identified sphingolipid metabolism as a key element regulating the phenotypes shift between TRCs and normal 2D−cultured cancer cells.

### 3.5. Alteration in Lipid Metabolism in PDAC TRCs via Lipidomic Analysis

**Figure 4:**
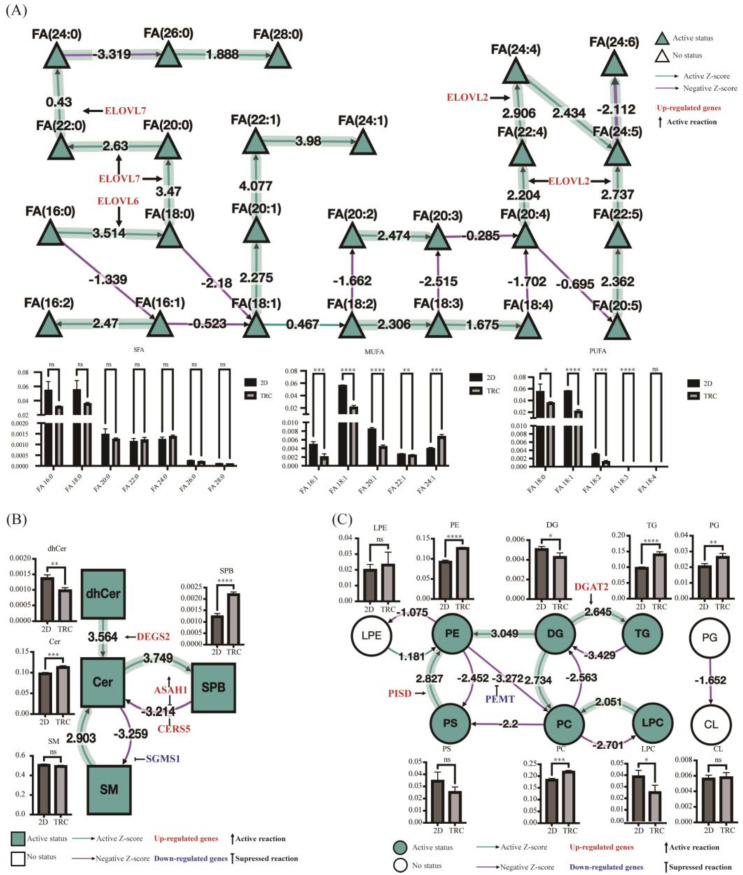
Lipid network generated using BioPAN software combined with alternation metabolites and related genes in PANC−1 TRCs compared to the normal 2D−cultured PANC−1 cells. (**A**) BioPAN fatty acids networks. FA graphs exported from BioPAN tool for PANC−1 TRCs compared to PANC−1. Green nodes correspond to active FAs and green shaded arrows to active pathways. Reactions with a positive Z score have green arrows, while negative Z scores are colored purple. Pathways options: PANC−1 TRCs condition of interest, PANC−1 control condition, lipid type, active status, subclass level, reaction subset of lipid data, *p* value 0.05, and no paired data. LMRGs of DEGs in red (up−regulated) of PANC−1 TRCs compared to PANC−1 cells using RNA−seq were consistent with the genes predicted as active (arrow) in BioPAN. (**B**,**C**) BioPAN lipid networks. Lipid network graphs exported from BioPAN for PANC−1 TRCs compared to PANC−1. Green nodes [glycerophospholipid metabolism in circle (**B**) and sphingolipid metabolism in square (**C**)] correspond to active lipids and green shaded arrows to active pathways. Reactions with a positive Z score have green arrows while negative Z scores are colored purple. Pathways options: PANC−1 TRCs condition of interest, PANC−1 control condition, lipid type, active status, subclass level, reaction subset of lipid data, *p* value 0.05, and no paired data. LMRGs of DEGs in red (up−regulated) or in blue (down−regulated) ofPANC−1 TRCs compared to PANC−1 cells using RNA−seq were consistent with the genes predicted as active (arrow) or suppressed (Long line truncated by short dash) in BioPAN. *, *p* < 0.05; **, *p* < 0.01; ***, *p* < 0.001; ****, *p* < 0.0001; ns, not significant; *t*-test. TRCs, tumor-repopulating cells; FA, fatty acid; MUFA, monounsaturated fatty acid; PUFA, polyunsaturated fatty acid; SFA, saturated fatty acid; Cer, ceramide; dhCer, dihydroceramide; SPB, sphingosine; SM, sphingomyelin; LPC, lysophosphatidylcholine; PE, phosphatidylethanolamine; PS, phosphoserine; LPE, lysophos-phatidylethanolamine; PC, phosphatidylcholine; CL, cardiolipin; TG, triglycerides; DG, diglycerides.

Due to the similarity between the enrichment results of differential LMRGs in PANC−1 and PANC−1 TRC and the results of common differential LMRGs in the two cell lines, lipidomic analysis based on LC-MS was performed in PANC−1 TRC and PANC−1 to explore the differences in lipid metabolism products and further understand the lipid metabolism characteristics of PDAC CSCs. Principal component analysis (PCA) revealed a difference in lipidome in two groups (Figure S4). Thirteen types of lipids, including 435 lipid metabolites, were detected. In addition to varying trends in fatty acids with different chain lengths, it was also found that sphingosine (SPB), ceramide (Cer), phosphatidylethanolamine (PE), phosphatidylcholine (PC), phosphatidylglycerol (PG), and triglycerides (TG) significantly increased in TRCs, while dihydroceramide (dhCer), diglycerides (DG), and lysophosphatidylcholine (LPC) significantly decreased in TRCs. The lipidomic analysis was performed via BioPAN [48], which combined current knowledge of lipid metabolism and predicted genes to compare two biological conditions to identify activated or suppressed pathways using Z-score values (Table S8). The results of fatty acid metabolism were showed in Figure 4A. In general, palmitic acid (FA 16:0) and stearic acid (FA 18:0) were the most common FA in PANC−1 and PANC−1 TRC. Meanwhile, the longer chain FA and the extremely long chain fatty acids (FA 24:1) were found significantly increased in PANC−1 TRC. Consistent with this finding, the BioPAN network map of FA metabolism showed that the elongation of FA was the most significantly activated pathway in the FA metabolism pathway, including the monounsaturated fatty acids (FA (18:1) → FA (20:1) → FA (22:1) → FA (24:1), Z-score = 5.965), saturated fatty acids (FA (16:0) → FA (18:0) → FA (20:0) → FA (22:0) → FA (24:0) → FA (26:0) → FA (28:0), Z-score = 3.516), and polyunsaturated fatty acids [FA (20:4) → FA (22:4) → FA (24:4) → FA (24:5) → FA (24:6), Z-score = 2.737]. In sphingolipids metabolism (Figure 4B), active reaction chains (dhCer → Cer → SPB, Z-score = 5.171; SM → Cer → SPB, Z-score = 4.704) and suppressed reaction chains (SPB → Cer → SM, Z-score = 4.577) jointly lead to a significant accumulation of sphingosine in PANC−1 TRC. The reaction chain of PE generated by DG and PS in the glycophoric metabolism reaction is activated, while the reaction chain of PE as a substrate (PE → PC → LPC, Z-score = 4.224, PE → PC → PS, Z-score = 3.869) is suppressed, leading to an increase in PE in PANC−1 TRC (Figure 4C). Furthermore, we validated and analyzed the predicted genes in the BioPAN analysis with RNA-seq data. It was found that these elongations of very-long-chain FA genes *ELOVL2*, *ELOVL6*, and *ELOVL7* were significantly overexpressed in TRC groups. DEGS2 actively catalyzing dhCer → Cer → SPB reaction chains, and CERS5 suppressing the generation of Cer by SPB, and ASAH1 involved in both reactions were significantly overexpressed. These results were consistent with the genes predicted as active or suppressed in BioPAN. The results of metabolite differences (Z-score) in lipidomic, and consistency between changes in metabolic genes and metabolites, highlighted that the up-regulation of the sphingolipid metabolism pathway played the most special role in the lipid metabolic remodeling process of PDAC TRCs.

### 3.6. Identification of SPHK1 as a Key Lipid-Metabolism-Related Stemness Gene in PDAC

**Figure 5:**
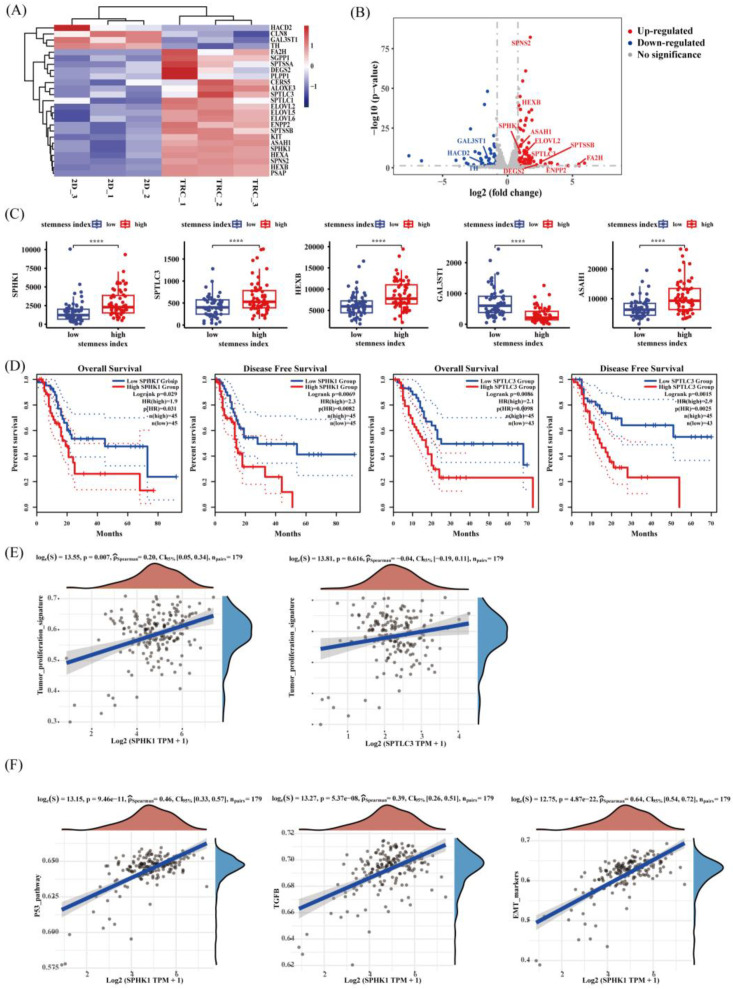
Identification of the key stemness LMRGs in PDAC. (**A**) Heatmap of gene expression (relative high expression in red and relative low expression in blue) which were enriched in sphingolipid metabolic process by GO enrichment analysis. (**B**) Volcano plot of the lipid-relative DEGs between PANC−1 TRC and PANC−1. Plots in red (up-regulated) or in blue (down-regulated) with gene names representing significant DEGs (|logFoldChange| > 1 and FDR < 0.05) enriched in sphingolipid metabolic process. (**C**) The correlation between the five genes (*SPHK1, SPTLC3, HEXB, GAL3ST1,* and *ASAH1*) and stemness indices by ssGSEA (****, *p* < 0.0001; *t*-test). (**D**) OS and DFS curves of PDAC patients from TCGA clustered by the expression of SPHK1 and SPTLC3 with quartile as group cutoff. (**E**) The correlation between the two genes (*SPHK1* and *SPTLC3*) and tumor proliferation signature using ssGSEA analysis. (**F**) The correlation between SPHK1 and TGF-beta, P53 pathways, and EMT markers using ssGSEA analysis (*n* = 179, Spearman correlation analysis). SPHK1, sphingosine kinases 1; PDAC, pancreatic ductal adenocarcinoma; GO, Gene Ontology; DEGs, differential expressed genes; SPTLC3, serine palmitoyltransferase 3; HEXB, beta-hexosaminidase; GAL3ST1, galac-tose-3-O-sulfotransferase 1; ASAH1, N-acylsphingosine amidohydrolase 1; ssGSEA, single sample gene set enrichment analysis; DFS, disease-free survival; OS, overall survival; TGF-beta, transforming growth factor-beta; EMT, epithelial mesenchymal transition.

Taking into account the comprehensive integrated transcriptomic and lipidomic analysis, the up-regulation of the sphingolipid metabolism pathway was found to be the most significant lipid metabolic remodeling process of PDAC TRCs. This finding was also supported by patient data analysis. The LMRGs in DEGs of PDAC patients in the high stemness group and in the low stemness group were enriched in the sphingolipid metabolism biological process via GO analysis and the sphingolipid metabolism pathway via KEGG analysis (Figure 1J,K). To identify the key genes of PDAC CSCs’ sphingolipid metabolism, the 25 common genes of sphingolipid metabolic process among GO enrichment results (Figure 5A and Figure S3A) were further screened using the thresholds (|logFoldChange| > 1 and FDR < 0.05). A total of 14 LMRGs were significantly differently expressed between PANC−1 TRC and PANC−1 (Figure 5B), and 8 of them were significantly different in the high stemness group and in the low stemness group, among which only the expression differences in *SPHK1*, *SPTLC3*, *HEXB*, *GAL3ST1*, and *ASAH1* were consistent in the CSLCs model and patients’ grouping by stemness indices (Figure 5C and Figure S5). Moreover, survival analysis (Figure 5D) showed that patients with high expression of SPHK1 suffered a shorter median OS (*p* = 0.029) and shorter median disease-free survival (DSS, *p* = 0.0069) than those with low expression, and that patients with *SPTLC3* high expressed had poor median OS (*p* = 0.0086) and median DFS (*p* = 0.0015). No significant survival difference was found to be in association with the expression of *HEXB*, *GAL3ST1*, and *ASAH1* (Figure S5B). The expression level of SPHK1 (*p* = 0.007) was significantly positively correlated with the tumor proliferation signature instead of *SPTLC3* (*p* = 0.616) using ssGSEA analysis (Figure 5E). In addition, a positive correlation between the expression of SPHK1 and malignant biological signaling pathways of CSCs including TGF-beta, P53 pathways, and EMT markers [49] was observed as well (Figure 5F). Accordingly, *SPHK1* was considered as a key LMRG involved in stemness and prognosis in PDAC.

### 3.7. SPHK1 Promotes the Malignant Behaviors of PDAC-TRC by Promoting Stemness

**Figure 6:**
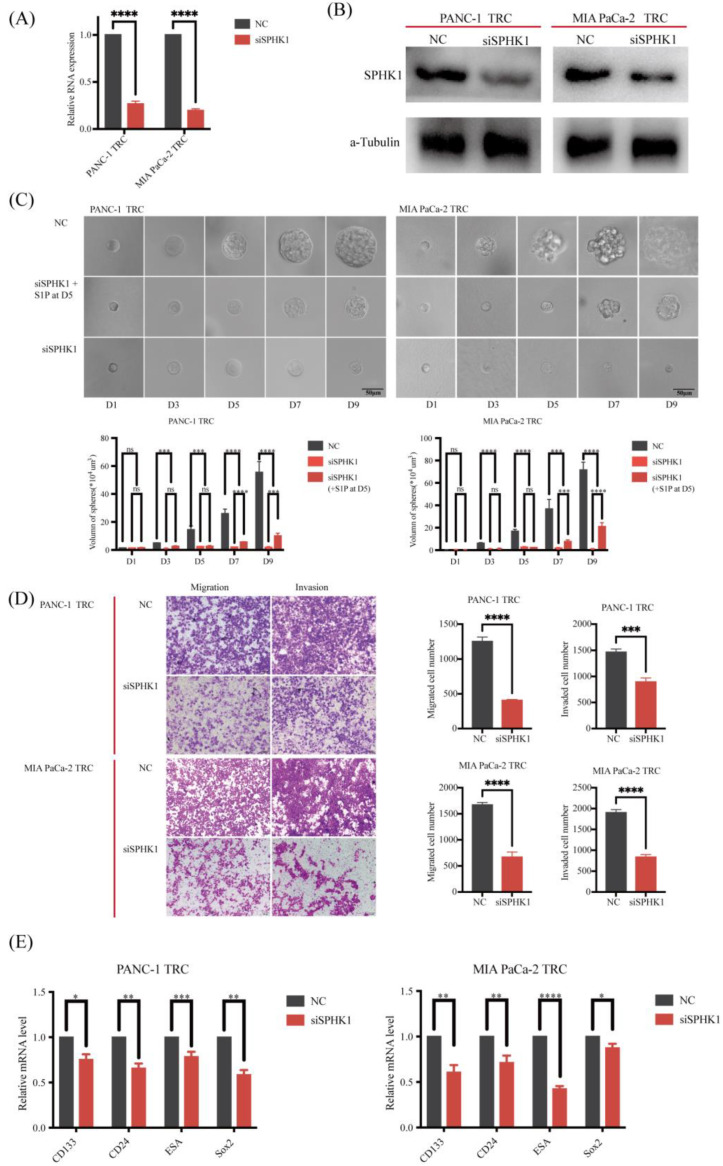
Effect of silencing SPHK1 by transfected siRNA to PDAC TRCs compared to the negative control (NC). (**A**) The mRNA level of SPHK1 in PDAC TRCs transfected with siSPHK1 and NC via qRT-PCR (mean ± SD, *n* = 3, *t*-test). (**B**) The expression of SPHK1 in PDAC TRCs transfected with siSPHK1 and NC via Western blotting. (**C**) Effect of silencing SPHK1 compared to NC on the colony growth of PDAC TRCs (mean ± SD, *n* = 3, *t*-test). (**D**) Effect of silencing SPHK1 compared to NC on the migration and invasion ability of PDAC TRCs via transwell assay (mean ± SD, *n* = 3, *t*-test). (**E**) Effect of silencing SPHK1 compared to NC on the expression of CSCs markers (ESA, CD133, Sox2 and CD24) detected via qRT-PCR (mean ± SD, *n* = 3, *t*-test). * *p* < 0.05; **, *p* < 0.01; ***, *p* < 0.001; and ****, *p* < 0.0001; ns, not significant. SPHK1, sphingosine kinases 1; PDAC, pancreatic ductal adenocarcinoma; TRCs, tumor-repopulating cells; qRT-PCR, quantitative reverse transcription polymerase chain reaction; S1P, Sphingosine 1-phosphate; CSCs, cancer stem cells; ESA, erythropoiesis-stimulating agent; Sox2, sex-determining region Y-box 2.

Finally, the biologic function of SPHK1 was evaluated in PDAC-TRC. The expression of SPHK1 was silenced using siRNA in PANC−1 TRC as well as MIA PaCa−2 TRC, being validated via qRT-PCR and Western blotting (Figure 6A,B). Silencing SPHK1 significantly inhibited the clonogenicity of both PANC−1 TRC as well as MIA PaCa−2 TRC (Figure 6C), and significantly decreased the migration and invasion ability of PANC−1 TRC as well as MIA PaCa−2 TRC (Figure 6D). By the fifth day of cultivation of PDAC TRCs transfected with siSPHK1, exogenous supplementation of S1P was performed, which recovered the clonogenic ability of TRCs. Exogenous supplementation of S1P to normal 2D−cultured PANC−1 and MIA PaCa−2 cells also resulted in enhanced migration and invasion (Figure S6). These results suggest that SPHK1 played a crucial role in promoting malignant behaviors in PDAC TRC. In addition, we evaluated the effect of SPHK1 on promoting stemness in PDAC TRC. Silencing SPHK1 significantly decreased the expression of multiple CSCs biomarkers, such as CD133, CD24, Nanog and Sox2 (Figure 6E), which were up-regulated in PDAC TRC compared to 2D−cultured PDAC cells. Taken together, SPHK1 may drive the malignant behaviors of PDAC-TRC by promoting stemness.

## 4. Discussion

CSCs are thought to contribute to tumor heterogeneity, which is an essential and distinct feature of PDAC. The stemness indices calculated using the ssGSEA algorithm rather than OCLR algorithm were applied in our study to describe and quantify CSCs. Transcriptome and lipidomic analysis on PDAC TRCs, proved to be an available CSCs model, found that the up-regulation of the sphingolipid metabolism pathway played the most special role in the lipid metabolic remodeling process. Finally, we identified *SPHK1* as the key stemness gene involved in sphingolipid metabolism. This understanding of CSCs and lipid metabolism reprogramming paved the way for developing novel therapeutic strategies of PDAC, and SPHK1 might be an appropriate target candidate.

CSC represents a small group of cells with infinite proliferative capacity, which is considered the main cause of metastasis and therapeutic resistance [38]. Therefore, the targeted eradication of CSCs will be an important progress in PDAC treatment. However, it how to best define CSCs and the extent to which different tumor types can develop to tumor mass are still controversial. Despite these controversies, increasing evidence suggests that stem-cell-associated features, often referred to as “stemness”, are biologically important in cancer [50], and are strongly related to poor outcomes in a wide variety of cancers [51,52]. An innovative OCLR on transcriptome was used to obtain the stemness indices (mRNAsi), which has been proven to stratify recognized undifferentiated BRCA, AML, and gliomas [39]. However, in our study, the stemness indices using OCLR failed to find an association with tumors’ undifferentiated state and patients’ outcomes. The possible reason may be related to Alex Miranda’s findings in reproducing the OCLR algorithm, as the OCLR algorithm precludes an unbiased assessment of the relationship between stemness and tumor immunity [38]. Therefore, we adopted the ssGSEA algorithm mentioned in Alex Miranda’ report to calculate the stemness indices. The results showed that higher stemness indices were correlated with more advanced clinical stages, a higher degree of oncogenic dedifferentiation, and worse outcomes, and that the classification of patients into high and low stemness group accordingly could be an independent prognostic predictor. Since the stemness indices using the ssGSEA algorithm can stratify recognized undifferentiated cancers, they were used to provide an approach to explore comprehensive lipid metabolism pathways on undifferentiated cancers in patients. Although it is currently unclear whether the stemness indices obtained from a large number of tumor samples represent a rare true CSCs population, our findings may advance the development for quantitating PDAC stemness, and provide a basis for the therapeutic targeting of the stemness phenotype itself.

In this study, we have mapped the specific lipid metabolism features of PDAC TRCs by combining the changes in lipid metabolites via lipidomic analysis and the expression of genes encoding metabolic enzymes via transcriptomic analysis. To understand the relationship of the stemness phenotype itself and the lipid metabolism, 3D soft fibrin gel [36] was used to culture PDAC TRCs by adjusting matrix stiffness, a significant physical property of ECM, which exerts a vital role in PDAC stemness regulation [53]. The results of malignant behaviors and overexpressed CSCs’ makers verified PDAC TRCs as an available CSLCs model. Of note, we not only showed the enhancement of fatty acid prolongation in PDAC CSCs consistent with previous studies [34,35], but also found the unique changes in sphingolipid metabolism in PDAC CSCs for the first time. Sphingolipids are not only important structural components of biological membranes, but also bioactive molecules that play a predominant role in signal transduction, cell growth, differentiation, and programmed cell death and thus affect tumor suppression or survival [54]. De novo sphingolipid begins with the condensation of serine and palmitoyl-CoA by serine palmitoyltransferase (SPT) to form dhCer, and endogenous ceramides synthesized after dihydroceramide desaturation by dihydroceramide desaturase (DES). Ceramide is also generated via sphingomyelin hydrolysis by sphingomyelinases (SMases) and via glucosylceramide breakdown. In addition, the salvage pathway for ceramide generation utilizes the recycling of sphingosine by CERS1–6 [55,56]. Ceramide is a core molecule in sphingolipids’ metabolism. Although cellular stress can induce the accumulation of ceramide and mediate cancer cell death [57], the active metabolism of ceramide has been confirmed in various tumors [58,59]. Ceramide can be converted to ceride-1-phosphate (C1P) [60] and SM [58], respectively, and is also utilized as a precursor for the generation of glycosphingolipids (GSL) including glucosylceramide and lactosylceramide [61]. Ceramide is hydroxylated by ceramidases (CDases) to yield sphingosine, which is phonologically late, by SPHK1 (also known as SK1) or SPHK2 (also known as SK2) to generate S1P [62]. In our study, we observed a slight increase in ceramide levels in TRCs, which may be related to the active metabolism of ceramide due to its role as a substrate for generation of sphingolipids with pro-survival functions. Different from the previous study’s results that the increasing GSL [61] and C1P [60] in PDAC contribute to malignant metastasis and tumor progression, the significantly elevated sphingosine was observed in PDAC TRCs. The accumulation of sphingosine was the result of the significantly inhibited salvage pathway with the significantly activated sphingosine generation pathway. In addition, our study found a significant decrease in dhCer.

It is hypothesized that CSCs, due to their unlimited proliferation, are in a long-term high demand for energy and cell division substances, resulting in significant changes in enzyme quantity instead of enzyme activity through which bio-reactions in the normal cells may be precisely regulated. Our results demonstrated that the significantly changing lipid reaction chain is coordinated with the trend of changes in key genes involved. For example, we observed the enhanced DEGS2's consumption amount of dhCer as reported in colorectal cancer [63], the significantly inhibited salvage pathway by overexpressed CERS5, and the activated sphingosine generation pathway by overexpressed ASAH1. Moreover, we found that *SPHK1*, the gene encoding the key enzyme catalyzing S1P from sphingosine, was observed to be overexpressed not only in PDAC TRCs but also in PDAC patients with high stemness indices, and overexpressed SPHK1 predicted worse prognosis of PDAC patients. The findings were consistent with the quantification of SPHK1 in PDAC specimens via immunohistochemistry, indicating high SPHK1 expression is independently associated with lymphatic invasion and unfavorable prognosis in PDAC patients [64]. Overexpression of SPHK1 facilitates the retention of endothelial progenitor cells at the progenitor stage [65] and promotes the proliferation of neural progenitor/stem cells [66]. And the involvement of the SPHK1 in CSC functioning has been recently investigated in several malignancies, including glioblastoma [67], melanoma [68], hepatocellular carcinoma [69], and breast adenocarcinoma [70]. Therefore, we hypothesized that SPHK1 plays an important role in maintaining the stemness of PDAC. The ssGSEA analysis demonstrated that the expression level of SPHK1 was significantly positively correlated with TGF-beta, P53, EMT, and tumor proliferation signals, in accordance with the results that SPHK1 are involved in CSCs markers expression, and the sphericity, migration, and invasion abilities of PDAC TRCs. Another study also demonstrated that SPHK1 upregulation may play a potential role in early neoplastic transformation of inflammatory lesions in long-standing chronic pancreatitis patients [71]. Mebendazolee was proved to be used as a potential therapeutic agent for treating PDAC, because it selectively inhibited SPHK1 more than SPHK2 and regulated the levels of sphingolipids [62]. In addition, the inhibitor of SPHK1 was reported to be effective in the combination treatment of PDAC [72], and can enhance the therapeutic effect of gemcitabine [73].

Nevertheless, limitations exist in this study. First, although this study has comprehensively considered the transcriptome of patients’ tumors and the transcriptome and lipidomic characteristics at the cell level, our findings still need to be further verified in preclinical models such as PDX or PDO considering the unique tumor microenvironment of PDAC. Secondly, it is necessary to further explore the stemness phenotype of PDAC by combining single cell sequencing or metabolomics, which can more accurately reflect the role of lipid metabolism in PDAC. Finally, the treatment of CSCs remains at the theoretical level; therefore, targeted treatment of SPHK1 or sphingolipid metabolism should be more considered in combination therapy for exploration.

## 5. Conclusions

In this study, we explored the lipid metabolism reprogramming pathway in PDAC with high or low stemness indices. The sphingolipid metabolism pathway was associated with tumor stemness and SPHK1 was found to play an important role in promoting stemness and malignant behaviors in PDAC-TRC. Furthermore, SPHK1 was strongly correlated with patients’ prognosis and a malignant-tumor-behavior-related signature in PDAC patients. These findings provide a novel strategy for targeting tumor lipid metabolism to inhibit CSCs in PDAC.

## Figures and Tables

**Table 1:** The sequences of all primers.

Name	Forward Primer	Reverse Primer
β-actin	CCACGAAACTACCTTCAACTCC	GTGATCTCCTTCTGCATCCTGT
Sox2	CCTACAGCATGTCCTACTCGCA	CTGGAGTGGGAGGAAGAGGTAAC
CD24	CTCCTACCCACGCAGATTTATTC	AGAGTGAGACCACGAAGAGAC
CD133	GTACAACGCCAAACCACGACT	CGCACACGCCACACAGTAA
ESA	CACCAGTCTTCTTACCAAACACG	AGTCCATTAGGCAGTATCTCCAAG
SPHK1	CAGCTCTTCCGGAGTCACGT	CGTCTCCAGACATGACCACCA

**Table 2:** Univariate and multivariate Cox regression analysis determined the independent prognostic role of stemness.

Variable		*n*	Univariate Cox Analysis			Multivariate Cox Analysis		
			HR	95% CI	*p*	HR	95% CI	*p*
Age	Old (> 65)	86	1			NA		
	Young (≤65)	78	0.775	0.506–1.190	0.241			
Sex	Female	89	1			NA		
	Male	75	0.799	0.523–1.220	0.300			
TNM Stage	I	20	1			NA		
	II	134	2.11	0.965–4.620	0.062			
	NA	8						
Grade	G1	29	1			1		
	G2	86	1.980	0.987–3.960	0.055	1.501	0.746–3.018	0.255
	G3/4	47	2.590	1.250–5.340	0.010 *	1.807	0.876–3.726	0.109
	Gx	2						
Lymph node stage	N0	45	1			1		
	N1/2	114	2.100	1.230–3.580	0.007 *	1.875	1.052–3.343	0.033 *
	Nx	5						
Tumor stage	T1/2	28	1			1		
	T3/4	134	2.020	1.040–3.930	0.038 *	1.237	0.597–2.563	0.567
	Tx	2						
Stemness index	High	82	1			1		
	Low	82	0.486	0.313–0.756	0.001 *	0.594	0.379–0.932	0.023 *

HR, hazard ratio; CI, confidence interval; NA, not available; * means *p* < 0.05.

**Table 3:** Comparison of subcutaneous tumor development between PANC−1 and PANC−1 TRCs in nude mice.

Number of Cells	PANC−1 TRCs	PANC−1 Cells
2 × 10^6^	100.0% (6/6)	66.7% (4/6)
2 × 10^5^	100.0% (6/6)	66.7% (4/6)
2 × 10^4^	83.3% (5/6)	0

## Data Availability

Human RNA-seq data were downloaded from TCGA (https://portal.gdc.cancer.gov/, accessed on 20 November 2022). Cancer metabolic gene sets and enrichment gene sets (c2.cp.kegg.v7.4.symbols and h.all.v7.5.1.symbols) were obtained from the MSigDB website. The obtained RNA-Seq raw data were uploaded to the Sequence Read Archive (SRA) database of the National Center for Biotechnology Information (NCBI) (https://www.ncbi.nlm.nih.gov/, accessed on 20 October 2023) with the accession number PRJNA-1020096.

## Supplementary Materials

The following supporting information can be downloaded at: https://www.mdpi.com/article/10.3390/metabo13111132/s1, Figure S1: Correlation between stemness indices by OCLR and clinical features in PDAC patients; Figure S2: Tumor bearing nude mice display and tumor volumes statistics; Figure S3: Supplementary enrichment analysis of PDAC TRCs’ RNA-seq; Figure S4: Principal component analysis (PCA) of lipid profile; Figure S5: Key stemness genes in PDAC; Figure S6: Effect of exogenous supplementation of S1P on the migration and invasion ability of PDAC; Figure S7: Identification of lipid metabolic pathways in tumor tissue compared to normal tissue; Figure S8: Classification of stemness by 33% percentile and 66% percentile stemness indices and subsequent features analysis in PDAC patients; Figure S9: The correlation between the key genes enriched in sphingolipid metabolism biological process and stemness indices by top/bottom 1/3; Table S1: Stemness indices by ssGSEA algorithms; Table S2: Stemness index by OCLR; Table S3: GO enrichment results of differential expressed LMRGs between patients with high and low stemness indices stratified by mean indices (*n* = 82); Table S4: KEGG enrichment results of differential expressed LMRGs between patients with high and low stemness indices stratified by mean indices (*n* = 82); Table S5: KEGG enrichment results of differential expressed LMRGs between PANC−1 TRC and PANC−1; Table S6: KEGG enrichment results of differential expressed LMRGs between MIA PaCa−2 TRC and MIA PaCa−2; Table S7: KEGG enrichment results of differential expressed LMRGs of set 4; Table S8: The lipidomic analysis by BioPAN. Table S9: KEGG analysis of differential expressed LMRGs between tumor and normal tissue; Table S10: KEGG enrichment results of differential expressed LMRGs between patients with high and low stemness indices by the top 1/3 and the bottom 1/3 of indices (*n* = 58); Table S11: Univariate and multivariate Cox regression analysis of patients with stemness indices in the bottom 1/3 and top 1/3. The differential analysis comparing tumor tissues and normal tissue of PDAC patients (Figure S7 and Table S9) were described in the supplementary methods and results parts, as well as the analysis based on the classification of stemness by 33% percentile and 66% percentile stemness indices in PDAC patients (Figures S8 and S9 and Tables S10 and S11).
