# Supplementary material for: Transcriptome and Lipidomic Analysis Suggests Lipid Metabolism Reprogramming and Upregulating SPHK1 Promotes Stemness in Pancreatic Ductal Adenocarcinoma Stem-like Cells
Source: Metabolites. 2023 Nov 4;13(11):1132. doi: 10.3390/metabo13111132 (PMC10673379; doi:10.3390/metabo13111132)
Supplement: Supplementary file 1 [file metabolites-13-01132-s001.zip › Supplymentary figures and material.pdf]

Supplementary materials

supplementary figures

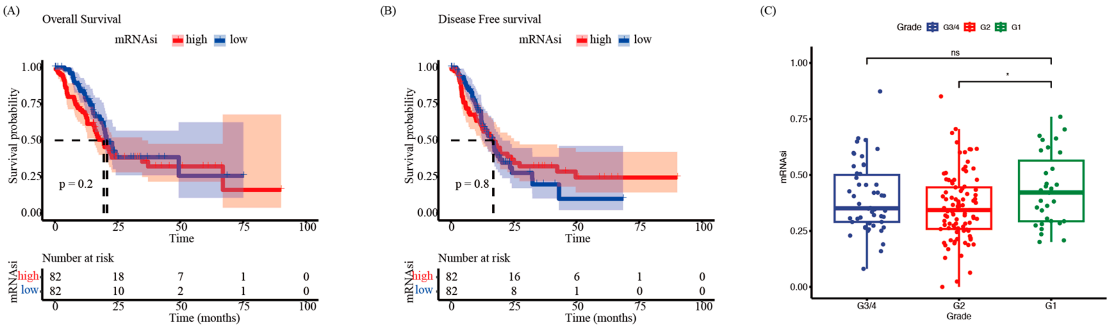

Figure S1. Correlation between stemness indices by OCLR and clinical features in PDAC patients. OS curve (A) and DFS curve (B) of patients with high and low mRNAsi by OCLR. (C) Boxplot of stemness indices by OCLR for PDAC patients stratified by histopathological grade. *, $P < 0.05$; ns, not significant; t-test. PDAC, pancreatic ductal adenocarcinoma; DFS, disease Free Survival; K-M curve, Kaplan-Meier curve; OS, overall survival.

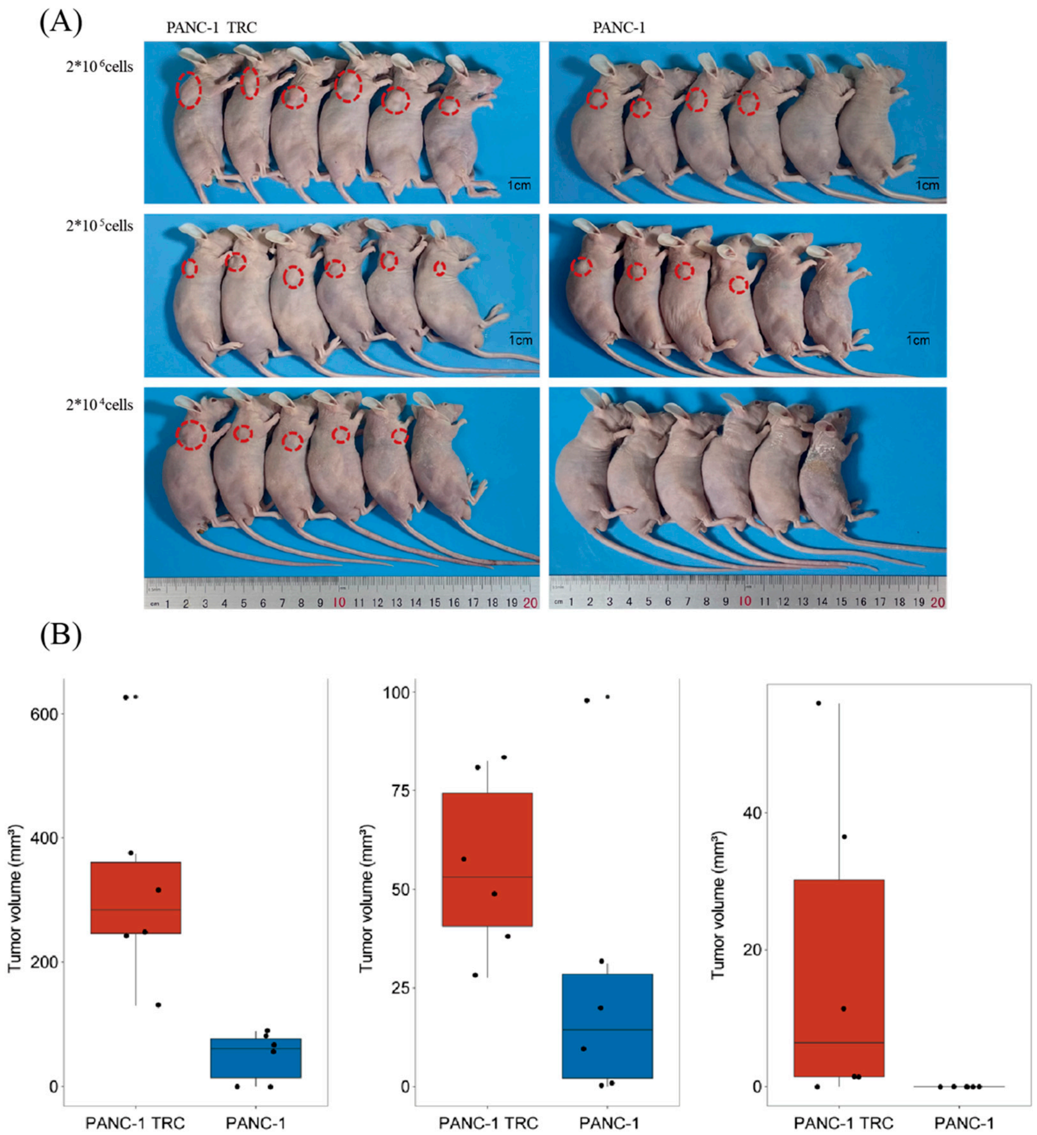

Figure S2. Tumor bearing nude mice display (A) and tumor volumes statistics (B). TRCs, tumor-repopulating cells.

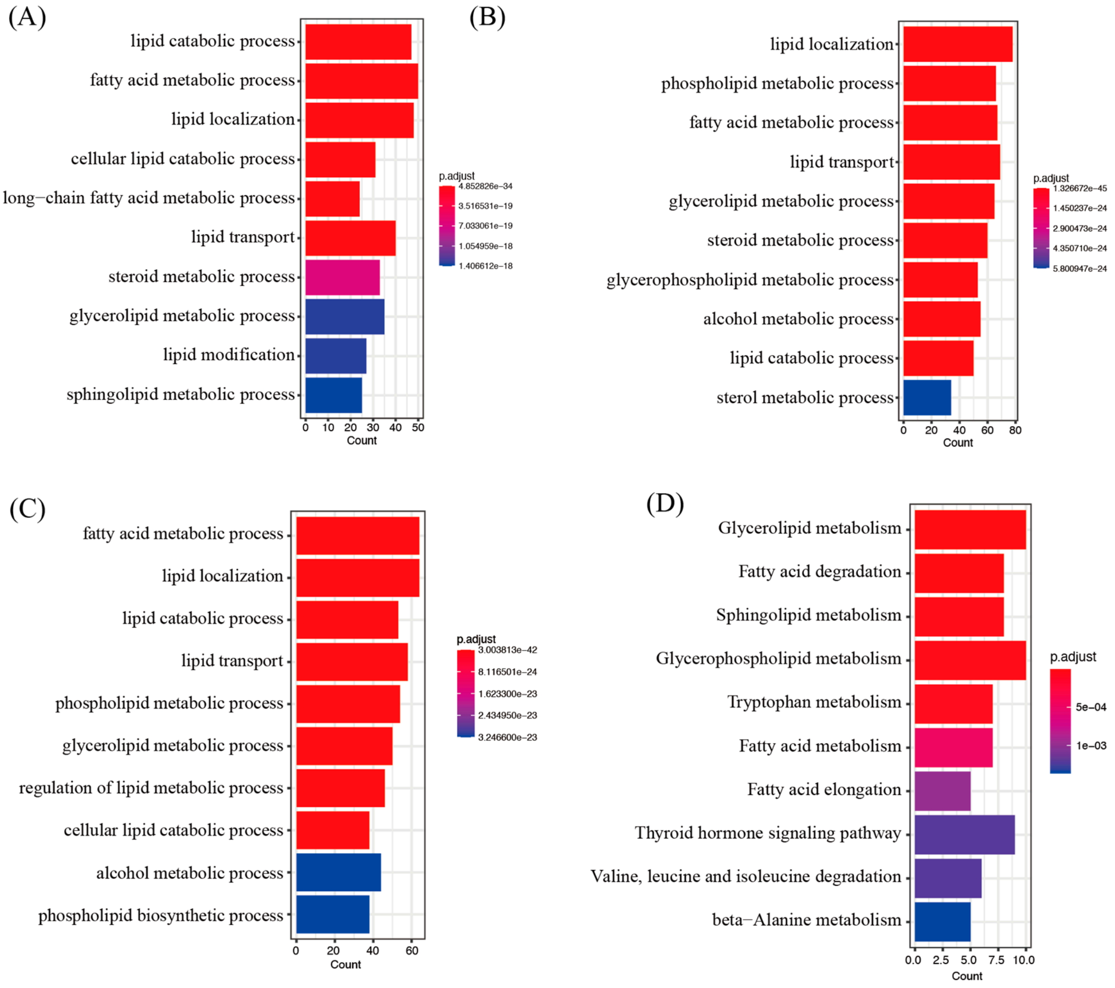

Figure S3. Supplementary enrichment analysis of PDAC TRCs' RNA-seq. (A) GO enrichment of set2 genes; (B) GO enrichment of set3 genes; (C) GO enrichment of genes with the same tendency in the two PDAC TRCs in set4; (D) KEGG enrichment of genes with the same tendency in the two PDAC TRCs in set4. PDAC, pancreatic ductal adenocarcinoma; TRCs, tumor-repopulating cells; DEGs, differential expressed genes; LMRGs, lipid metabolism-related genes; GO, Gene Ontology; KEGG, Kyoto Encyclopedia of Genes and Genomes.

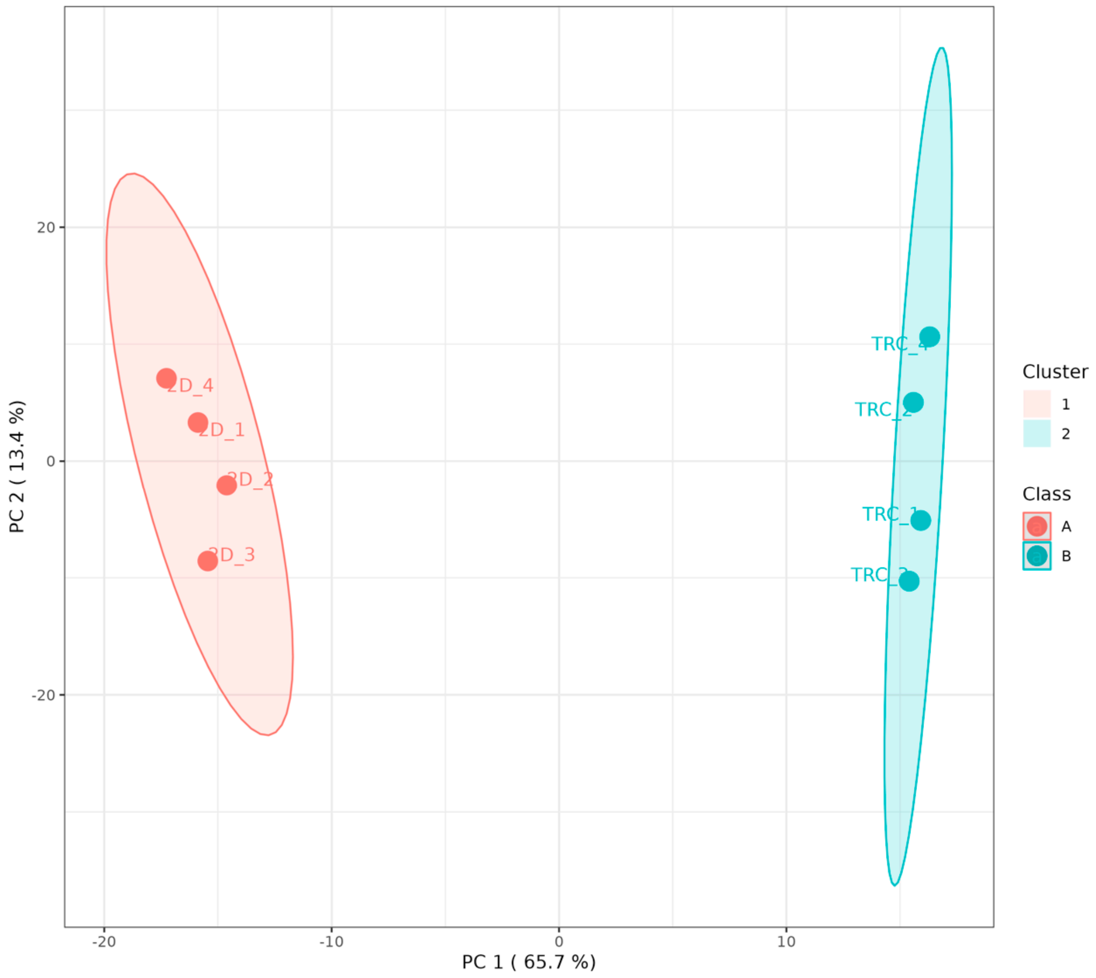

PC 2 (13.4 %)
PC 1 (65.7 %)
20
0
-20
-20
-10
0
10
2D_4
2D_1
3D_2
3D_3
TRC_4
TRC_2
TRC_1
TRC_3
Cluster
1
2
Class
A
B

Figure S4. Principal component analysis (PCA) of lipid profile.

(A)

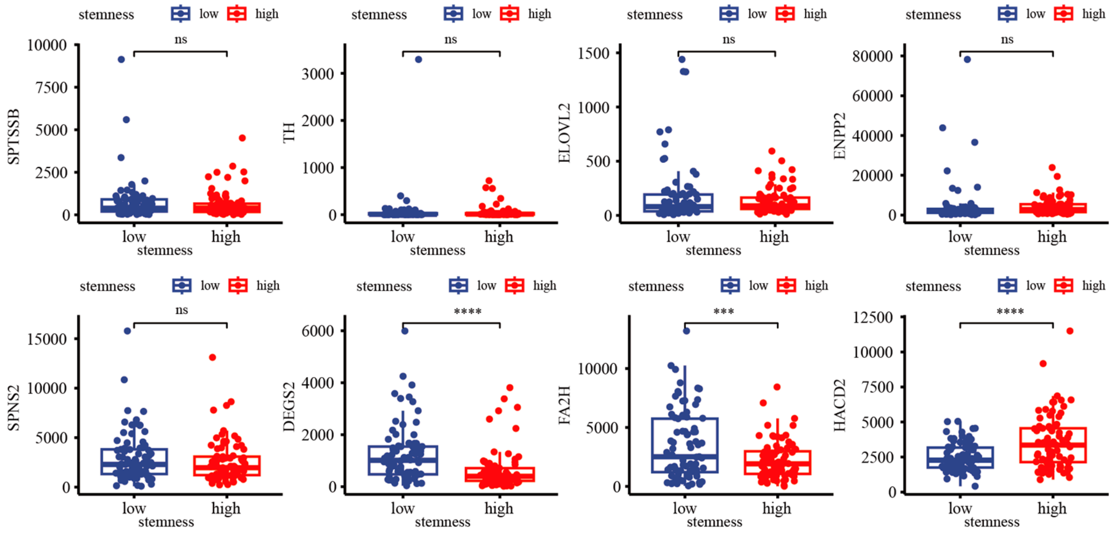

(B)

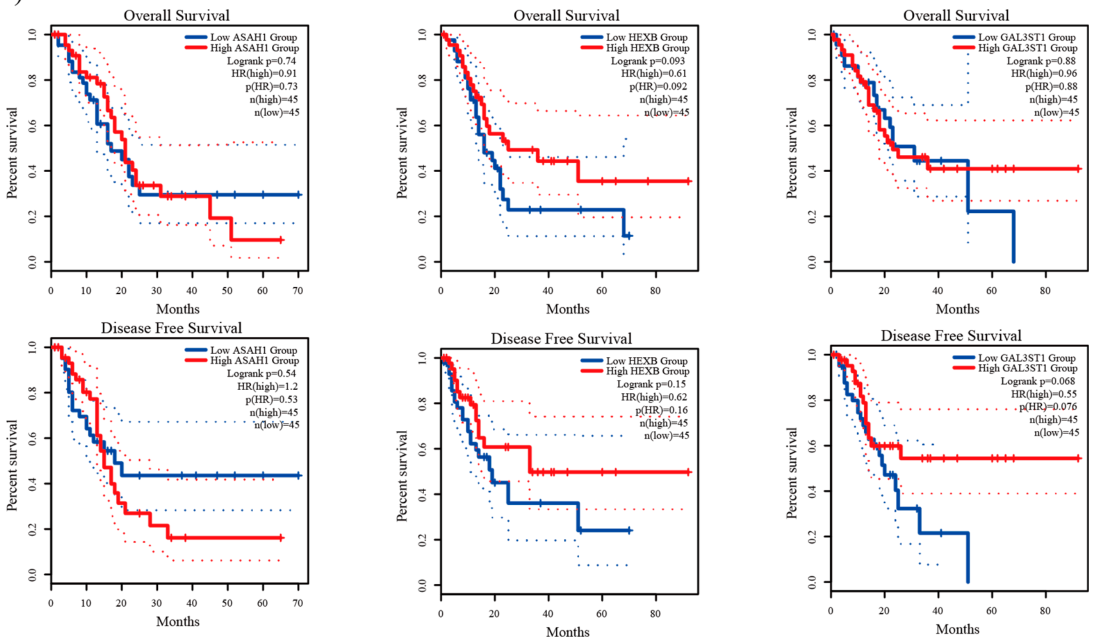

Figure S5. Key stemness genes in PDAC. (A) The correlation between the 8 genes (*SPTSSB, TH, ELOVL2, ENPP2, HACD2, FA2H, DEGS2, SPNS2*) and stemness indices by ssGSEA. (***, p<0.001; and ****, p<0.0001; ns, no significance; t-test) (B) OS and DFS curves of PDAC patients from TCGA clustered by the expression of *HEXB*, *GAL3ST1*, and *ASAH1*with quartile as group cutoff. PDAC, pancreatic ductal adenocarcinoma; HEXB, be-ta-hexosaminidase; GAL3ST1, galactose-3-O-sulfotransferase 1; ASAH1, N-acylsphingosine amidohydrolase 1; DFS, disease Free Survival; OS, overall survival.

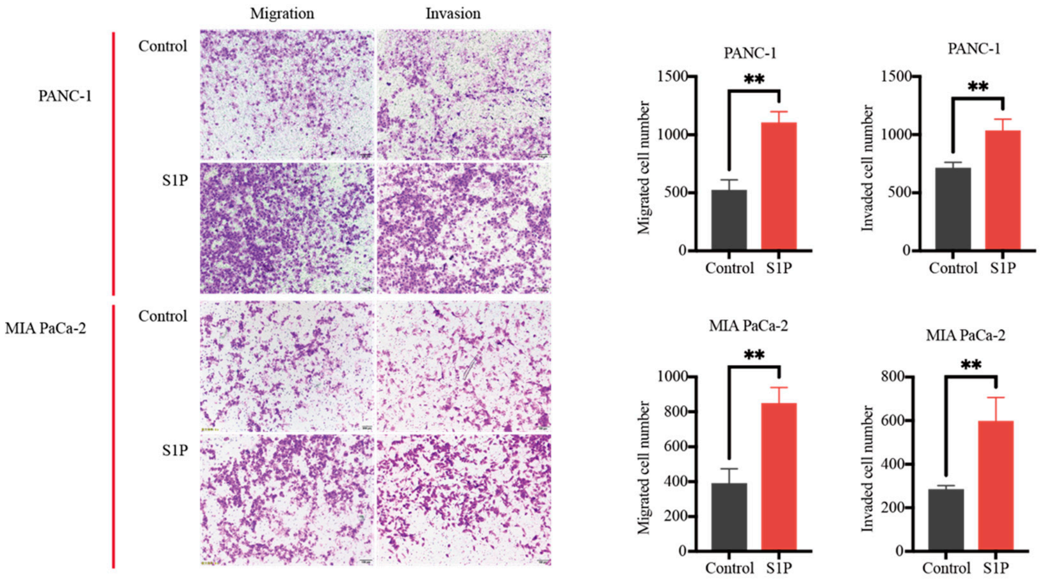

Figure S6. Effect of exogenous supplementation of S1P compared to NC on the migration and invasion ability of PDAC normal cells by transwell assay (mean ± SD, n = 3, t-test). **, $p<0.01$. S1P, Sphingosine 1-phosphate.

1. Supplementary methods

1.1. Patients' data collection and analysis

The comparison of tumor and normal tissue including genes' expression and patients' survival were conducted and plotted using Gene Expression Profiling Interactive Analysis 2 (GEPIA2.0, http://gepia2.cancer-pku.cn) with the mRNA expression profiles from TCGA data portal (tumor, n=179, normal, n=4) and normal pancreas mRNA expression data from GTEx (Genotype-Tissue Expression) portal (normal, n=167). The GEPIA2 website downloaded the TCGA and GTEx isoform expression data that are re-computed from raw RNA-Seq data by the UCSC Xena project based on a uniform pipeline[38]. PDAC patients from TCGA data portal (tumor, n=179) were arranged according to the stemness indices from low to high, and patients with stemness indices in the first 1/3 and last 1/3 were classified into the low stemness group and the high stemness group, respectively (Figure S8A).

2. Supplementary results

2.1. Differential analysis comparing tumor tissues and normal tissue.

Differential analysis comparing tumor tissues (n=179) and normal tissue (n=171) identified a set of differentially expressed genes (DEGs), about 3.1% (286/9219) of which were in-volved in lipid metabolism pathways (Figure S7A). To understand the characteristics of lipid metabolism of PDAC, GO and KEGG enrichment analysis (Figure S7B-C) were performed and several specific genes and lipid metabolic pathways were identified. The most significant difference in lipid metabolism pathways between tumors and normal tissues were arachidonic acid metabolism, steroid hormone biosynthesis, and fat digestion and absorption (Table S9).

2.2. Classification of stemness by 33% percentile and 66% percentile stemness indices and subsequent features analysis in PDAC patients

Patients with stemness indices in the bottom 1/3 and top 1/3 were classified into the low stemness group (n = 58) and the high stemness group (n = 58). The K-M curve results showed that patients in the high stemness group suffered shorter median-OS (high stemness group vs low stemness group, 16.4 vs 24.1 months, P = 0.0024) and median-PFS (high stemness group vs low stemness group, 12.4 vs 42.3 months, P = 0.0027). Cox multivariate analysis with significant factors obtained from the univariate analysis (P < 0.05) was carried out to further assess the relationship between tumor stemness and patients' OS (Table S11) and it was found that patients in low stemness group was an independent favorable prognosis factor for PDAC (HR=0.535, 95% CI, 0.316-0.905, P=0.020). GO and KEGG enrichment analysis (Figure 1J-K, Table S10) were performed and several specific genes and lipid metabolic pathways were identified. Moreover, when identifying the key genes of PDAC CSCs' sphingolipid metabolism, the same genes including SPHK1, SPTLC3, HEXB, GAL3ST1, and ASAH1 were consistent in CSLCs model and patients' grouping (Figure S9) by stemness indices.

Table S11. Univariate and multivariate Cox regression analysis of patients with stemness indices in the bottom 1/3 and top 1/3.

Variable		n	Univariate cox analysis			Multivariate cox analysis		
			HR	95% CI	P	HR	95% CI	P
Age	Old	59	1			NA		
	Young	57	0.722	0.437-1.190	0.202			
Sex	Female	54	1			NA		

	Male	62	0.949	0.583-1.550	0.834			
TNM Stage	I	16	1			1		
	II	98	2.440	1.030-5.750	0.042 *	0.974	0.156-6.067	0.977
Grade	G1	26	1			1		
	G2	52	2.220	1.040-4.740	0.040 *	1.594	0.722-3.516	0.248
	G3/4	38	3.200	1.470-6.950	0.003 *	2.347	1.070-5.151	0.033 *
Lymph node stage	N0	30	1			1		
	N1	82	1.840	1.010-3.340	0.047 *	1.454	0.679-3.115	0.336
Tumor stage	T1/2	21	1			1		
	T3/4	93	2.220	1.040-4.710	0.038 *	1.076	0.251-4.614	0.921
Stemness index	High	58	1			1		
	Low	58	0.466	0.281-0.772	0.003 *	0.535	0.316-0.905	0.020 *

HR, hazard ratio; CI, confidence interval; NA, not available; * means P < 0.05.

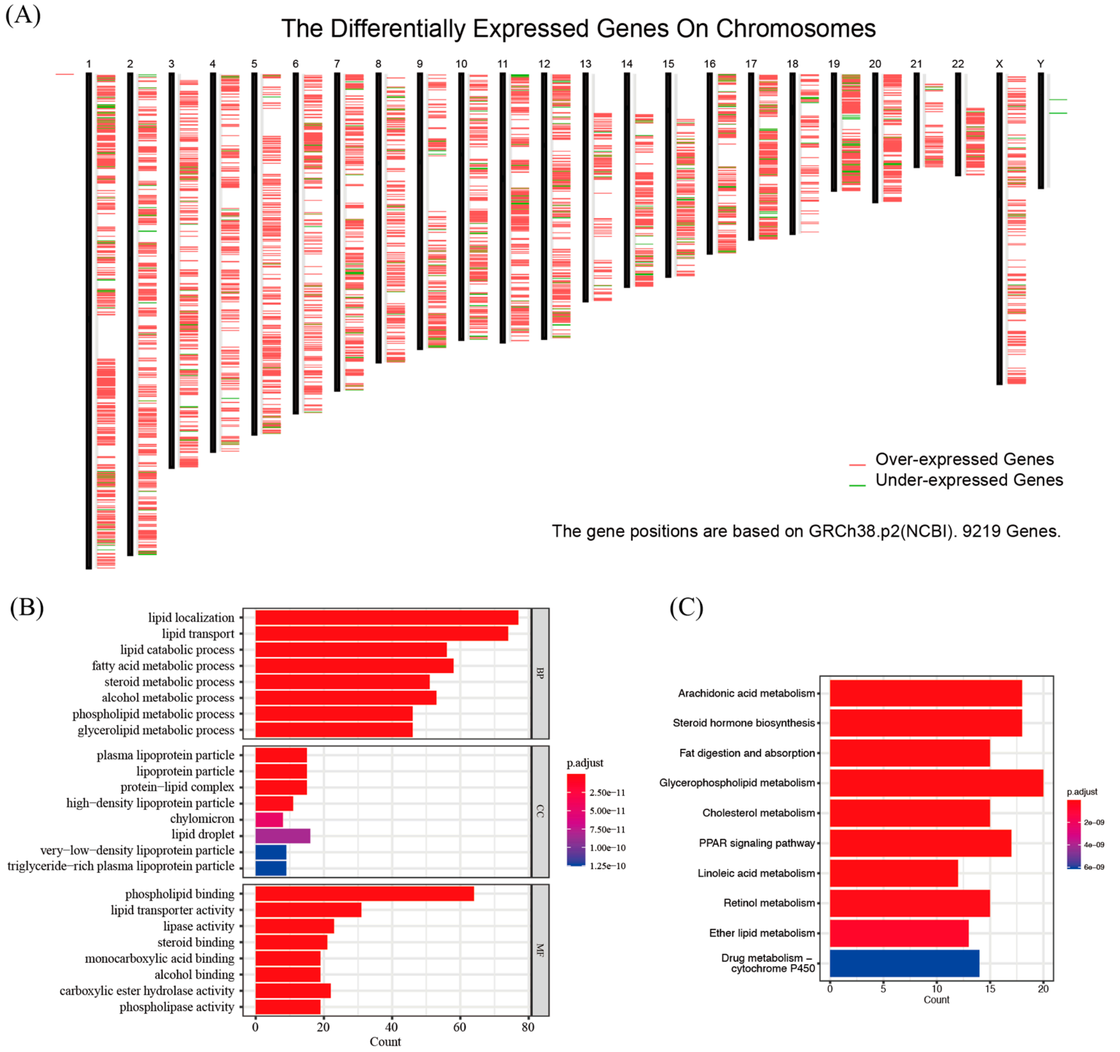

Figure S7. Identification of lipid metabolic pathways in tumor tissue compared to normal tissue. (A) DEGs in TCGA dataset (TCGA dataset, tumor, n = 179, normal, n = 4; GETx, normal, n = 167); (B) GO analysis of the differential expressed LMRGs in TCGA dataset. (C) KEGG analysis of the differential expressed LMRGs in TCGA dataset. DEGs, differential expressed genes; LMRGs, lipid metabolism-related genes; GO, Gene Ontology; KEGG, Kyoto Encyclopedia of Genes and Genomes.

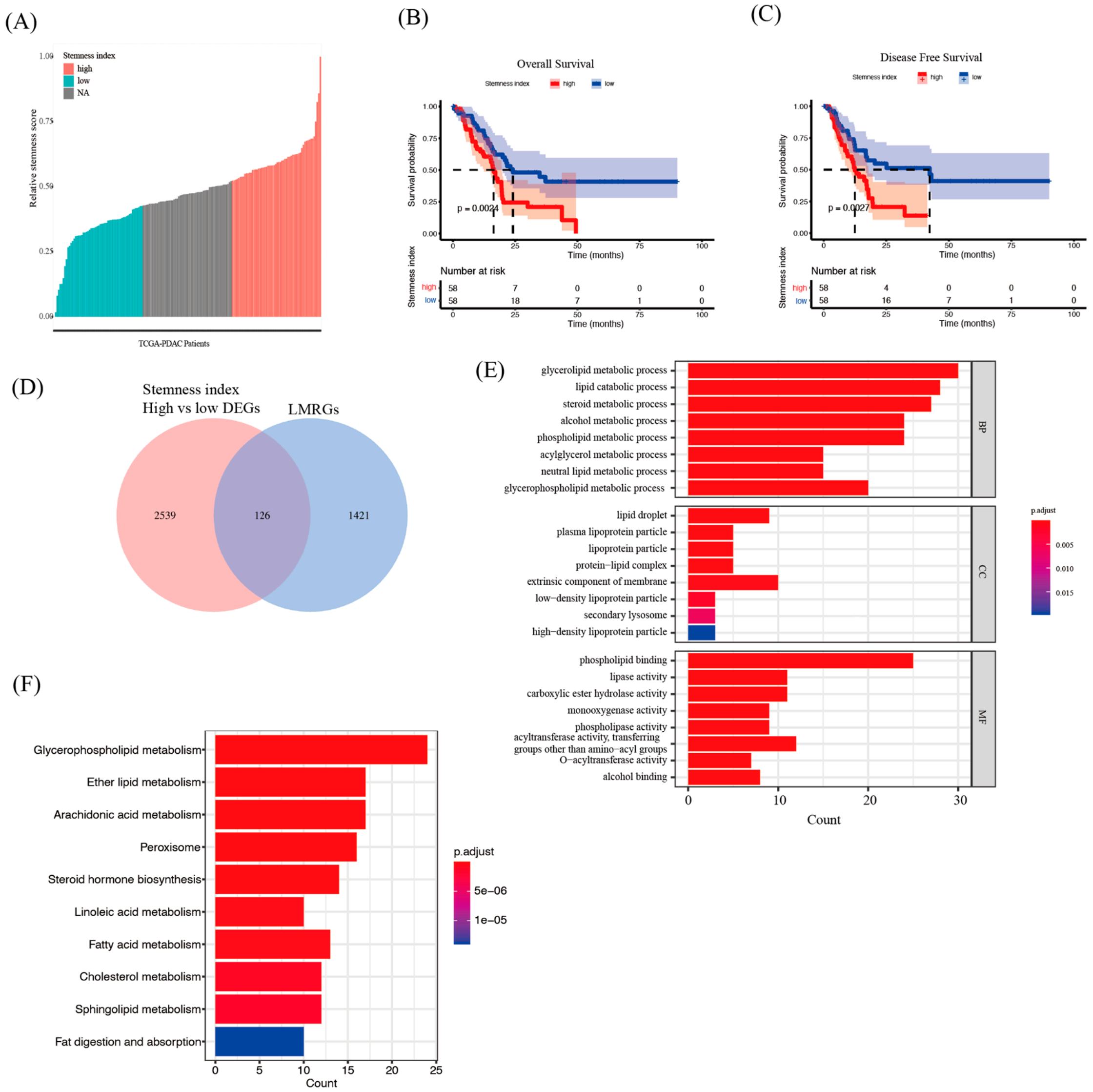

Figure S8. Classification of stemness by 33% percentile and 66% percentile stemness indices and subsequent features analysis in PDAC patients. (A) An overview of the distribution of relative stemness indices in PDAC patients (n = 179) and the classification of stemness groups (top 1/3 stemness indices as high stemness, n = 58; bottom 1/3 stemness indices as low stemness, n = 58). OS K-M curve (B) and DFS K-M curve (C) showed the outcomes of PDAC patients in high stem-ness group and low stemness group. (D) Venn diagram shows the overlapped genes between LMRGs and DEGs of the two stemness groups. (E) GO and (F) KEGG enrichment analysis of the overlapped genes. *, $p < 0.05$; and **, $p < 0.01$; Studen's t-test. ssGSEA, single sample gene set enrichment analysis; PDAC, pancreatic ductal adenocarcinoma; DFS, disease Free Survival; K-M curve, Kaplan-Meier curve; OS, overall survival; LMRGs, lipid metabolism-related genes; DEGs, differential expressed genes; GO, Gene Ontology; KEGG, Kyoto Encyclopedia of Genes and Genomes.

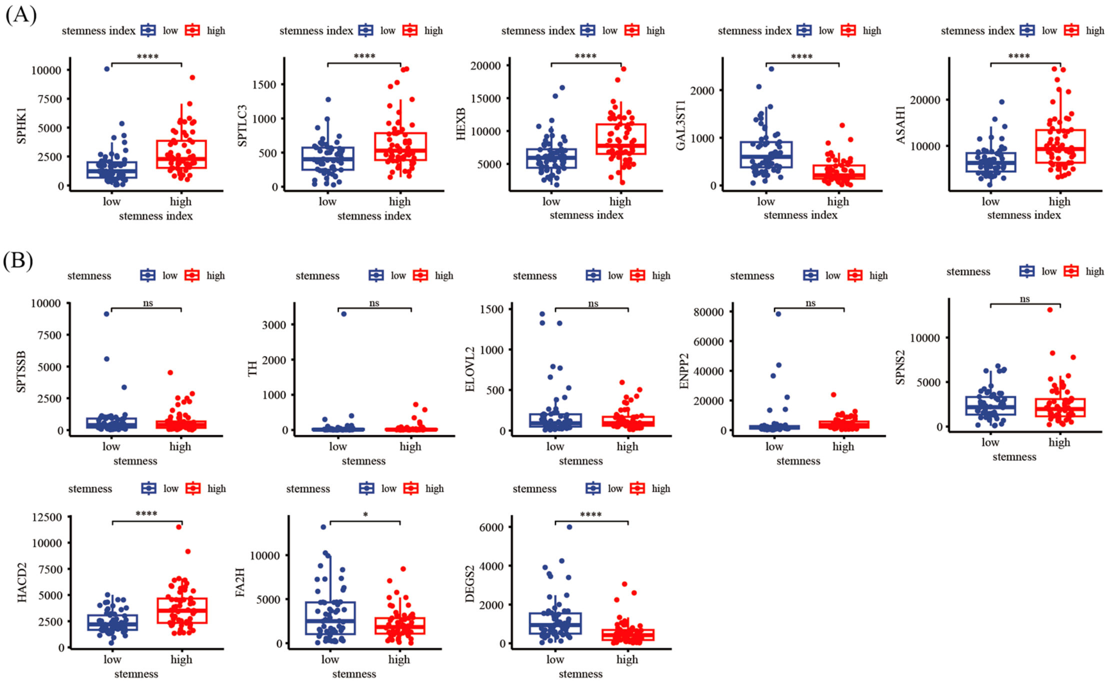

Figure S9. The correlation between the key genes enriched in sphingolipid metabolism biological process and stemness indices by top/bottom 1/3. **, $p<0.01$; ***, $p<0.001$; and ****, $p<0.0001$; t-test.
